# Palmitic acid control of ciliogenesis modulates insulin signaling in hypothalamic neurons through an autophagy-dependent mechanism

**DOI:** 10.1038/s41419-022-05109-9

**Published:** 2022-07-28

**Authors:** Yenniffer Ávalos, María Paz Hernández-Cáceres, Pablo Lagos, Daniela Pinto-Nuñez, Patricia Rivera, Paulina Burgos, Francisco Díaz-Castro, Michelle Joy-Immediato, Leslye Venegas-Zamora, Erik Lopez-Gallardo, Catalina Kretschmar, Ana Batista-Gonzalez, Flavia Cifuentes-Araneda, Lilian Toledo-Valenzuela, Marcelo Rodriguez-Peña, Jasson Espinoza-Caicedo, Claudio Perez-Leighton, Cristina Bertocchi, Mauricio Cerda, Rodrigo Troncoso, Valentina Parra, Mauricio Budini, Patricia V. Burgos, Alfredo Criollo, Eugenia Morselli

**Affiliations:** 1 Departamento de Biología, Facultad de Química y Biología, Universidad de Santiago de Chile, Santiago, Chile; 2 Laboratory of Autophagy and Metabolism, Department of Physiology, Faculty of Biological Sciences, Pontificia Universidad Católica de Chile, Santiago, Chile; 3 Cellular and Molecular Biology Laboratory, Institute in Dentistry Sciences, Dentistry Faculty, Universidad de Chile, Santiago, Chile; 4 Laboratory for Molecular Mechanics of Cell Adhesion, Pontificia Universidad Católica de Chile, Santiago, Chile; 5 Advanced Center for Chronic Diseases (ACCDiS), Faculty of Chemical and Pharmaceutical Sciences and Faculty of Medicine, Universidad de Chile, Santiago, Chile; 6 Department of Physiology, Faculty of Biological Sciences, Pontificia Universidad Católica de Chile, Santiago, Chile; 7 Integrative Biology Program, Institute of Biomedical Sciences, Facultad de Medicina, Universidad de Chile, Santiago, Chile; 8 Center for Medical Informatics and Telemedicine, Facultad de Medicina, Universidad de Chile, Santiago, Chile; 9 Biomedical Neuroscience Institute, Santiago, Chile; 10 Instituto de Nutrición y Tecnología de los Alimentos (INTA), Universidad de Chile, Santiago, Chile; 11 Autophagy Research Center, Santiago, Chile; 12 Network for the Study of High-Lethality Cardiopulmonary Diseases (REECPAL), Universidad de Chile, Santiago, Chile; 13 Laboratory of Molecular and Cellular Pathology, Institute in Dentistry Sciences, Dentistry Faculty, Universidad de Chile, Santiago, Chile; 14 Centro de Biología Celular y Biomedicina (CEBICEM), Facultad de Medicina y Ciencia, Universidad San Sebastián, Santiago, Chile; 15 Centro de Envejecimiento y Regeneración (CARE-UC), Facultad de Ciencias Biológicas, Pontificia Universidad Católica de Chile, Santiago, Chile; 16 Department of Basic Sciences, Faculty of Medicine and Sciences, Universidad San Sebastián, Santiago, Chile

**Keywords:** Insulin signalling, Macroautophagy, Mechanisms of disease, Cilia

## Abstract

Palmitic acid (PA) is significantly increased in the hypothalamus of mice, when fed chronically with a high-fat diet (HFD). PA impairs insulin signaling in hypothalamic neurons, by a mechanism dependent on autophagy, a process of lysosomal-mediated degradation of cytoplasmic material. In addition, previous work shows a crosstalk between autophagy and the primary cilium (hereafter cilium), an antenna-like structure on the cell surface that acts as a signaling platform for the cell. Ciliopathies, human diseases characterized by cilia dysfunction, manifest, type 2 diabetes, among other features, suggesting a role of the cilium in insulin signaling. Cilium depletion in hypothalamic pro-opiomelanocortin (POMC) neurons triggers obesity and insulin resistance in mice, the same phenotype as mice deficient in autophagy in POMC neurons. Here we investigated the effect of chronic consumption of HFD on cilia; and our results indicate that chronic feeding with HFD reduces the percentage of cilia in hypothalamic POMC neurons. This effect may be due to an increased amount of PA, as treatment with this saturated fatty acid in vitro reduces the percentage of ciliated cells and cilia length in hypothalamic neurons. Importantly, the same effect of cilia depletion was obtained following chemical and genetic inhibition of autophagy, indicating autophagy is required for ciliogenesis. We further demonstrate a role for the cilium in insulin sensitivity, as cilium loss in hypothalamic neuronal cells disrupts insulin signaling and insulin-dependent glucose uptake, an effect that correlates with the ciliary localization of the insulin receptor (IR). Consistently, increased percentage of ciliated hypothalamic neuronal cells promotes insulin signaling, even when cells are exposed to PA. Altogether, our results indicate that, in hypothalamic neurons, impairment of autophagy, either by PA exposure, chemical or genetic manipulation, cause cilia loss that impairs insulin sensitivity.

## Introduction

The global prevalence of obesity has more than doubled since 1980; and currently, more than 13% of the world adult population is obese [1]. One of the main factors that increase obesity rates is consumption of the so-called western style high-fat diet (HFD), a diet rich in saturated fatty acids (SatFAs), mainly palmitic acid (PA), which promotes the development of obesity and obesity-associated metabolic diseases such as insulin resistance [2, 3].

It is widely accepted that SatFAs decrease insulin sensitivity in several peripheral tissues as well as in the central nervous system (CNS), specifically in the hypothalamus, a key brain site involved in the regulation of peripheral insulin sensitivity and glucose homeostasis [4, 5]. PA is significantly increased in the plasma of obese humans [6] and in the hypothalamus of mice following chronic HFD consumption [7, 8], promoting insulin resistance by mechanisms that have been only partially unveiled.

In the hypothalamus, neurons and astrocytes, but not microglia, have a structure, similar to an antenna, known as the primary cilium (hereafter cilium) [9, 10]. The cilium is a non-motile and solitary (one per cell) organelle composed of a ciliary membrane that surrounds a microtubule-based axoneme, which nucleates from the basal body [11]. The microtubules of the axoneme serve as tracks for intraflagellar transport (IFT) during its assembly and provide the scaffold for the binding of various protein complexes. IFT particles and their associated cargo proteins are transported in an anterograde direction to the tip of the cilium by kinesin-2 motor proteins [12]. Kinesin Family Member 3A (KIF3A), a kinesin-2 family motor protein, together with KIF3B and kinesin-associated protein (KAP), form a heterotrimeric motor for IFT along the ciliary axoneme, thus allowing the growth of the cilium [13, 14]. Depletion of IFT88 or KIF3A disrupts the cilium and therefore its function [12, 15]. The ciliary membrane is enriched in receptors that allow the cell to sense, transduce, and integrate a variety of extracellular stimuli, thus the cilium is considered as a signaling platform [16]. Interestingly, the presence and the location of these receptors within the cilium change in response to different stimuli and in response to the metabolic status of the cell [17]. A subgroup of patients with ciliary dysfunctions are obese and present metabolic syndrome [18]. Human genetic ciliopathies such as Bardet–Biedl syndrome and Alström’s syndrome manifest obesity and type 2 diabetes [19, 20]. Inhibition of ciliogenesis in anorexigenic proopiomelanocortin (POMC) hypothalamic neurons, in neonatal mice, leads to adult obesity [21]. The same obesity-prone phenotype is shown in mice, when the autophagic process is inhibited in POMC neurons [22–25].

Macroautophagy, hereafter autophagy, is a key process for the maintenance of cellular and tissue homeostasis through the turnover of cytoplasmic material. Growing evidence supports a bidirectional relationship between ciliogenesis and autophagy [26]. Autophagy has been demonstrated to promote ciliogenesis in mouse embryonic fibroblasts (MEF) and human retinal pigmented epithelial (RPE) cells [27], by degrading ciliogenesis inhibitory proteins [28]. The pharmacological or genetic ablation of autophagy decreases the percentage of ciliated cells and cilium length in the same models [26]. Conversely, the primary cilium can regulate autophagy through modulation of suppression of mTORC1 activity [28]. These data suggest that proper autophagic function is necessary to adequate cilia function, and vice versa.

We and others have shown that autophagy regulates insulin signaling [29–31]. We demonstrated that genetic and chemical inhibition of autophagy in hypothalamic neuronal cells reduces insulin signaling, finally preventing insulin-dependent glucose uptake [31], by mechanisms that have not yet been unveiled. Interestingly, the insulin receptor (IR) has been identified in the primary cilium of pancreatic β-cells [32], suggesting the possible involvement of the IR at the cilium of hypothalamic neurons.

Here we show that chronic consumption of a pro-obesogenic HFD, which promotes the accumulation of SatFAs and inhibits the autophagic flux in the hypothalamus [7, 8, 33, 34], reduces the percentage of ciliated hypothalamic POMC neurons. Treatment of hypothalamic neurons with PA, which as we previously showed blocks autophagy [31, 35], reduces the percentage of ciliated cells and cilia length. The same effect was obtained following chemical and genetic inhibition of autophagy. Our findings show that the primary cilium, in hypothalamic neuronal cells, is necessary and sufficient to regulate insulin signaling and insulin-dependent glucose uptake, an effect that correlates with the ciliary localization of the insulin receptor.

Altogether, these results suggest that insulin resistance caused by HFD feeding or PA, which inhibits autophagy, might be due to impaired ciliogenesis and loss of ciliary-dependent activation of IR-dependent signaling in hypothalamic neurons.

## Results

### Chronic HFD consumption, that induces obesity, promotes cilia loss in hypothalamic POMC neurons

**Fig. 1:**
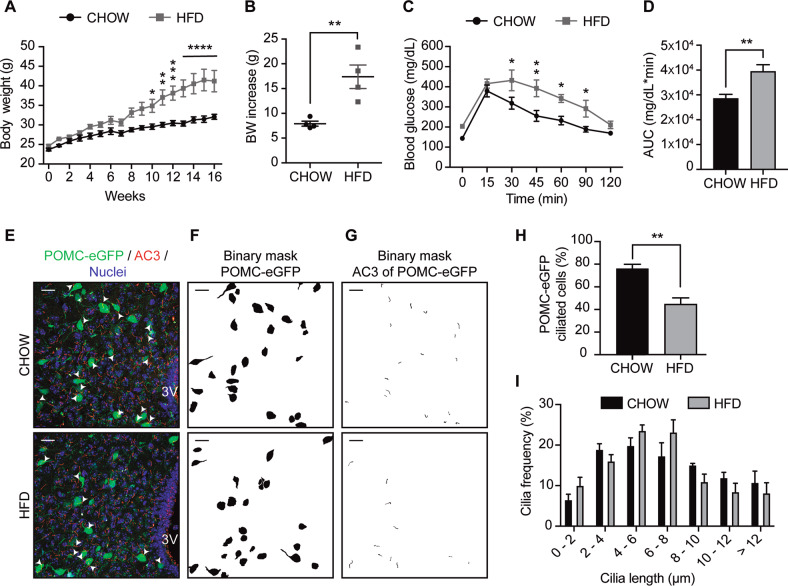
Chronic HFD consumption decreases the percentage of ciliated cells in hypothalamic POMC neurons. **A** Body weight of C57BL/6 transgenic mice expressing eGFP in POMC neurons (POMC-eGFP) on a chow or high-fat diet (HFD) for 16 weeks. **B** Body weight (BW) increase of POMC-eGFP mice fed chow or HFD after 16 weeks. **C** Glucose tolerance test (GTT) and **D** area under the curve (AUC) of GTT of POMC-eGFP mice fed chow or high-fat diet (HFD) for 16 weeks. **E** Representative confocal images showing POMC-eGFP neurons and AC3 (adenylate cyclase 3, cilia) immunoreactivity in the arcuate nucleus of the hypothalamus, with the respective binary masks (**F**, **G**) of mice fed chow or HFD for 16 weeks. Arrowheads indicate cilia of POMC-eGFP neurons. Scale bar: 20 µm. Nuclei were stained with Hoechst (blue). **H** Percentage of ciliated cells in POMC-eGFP neurons. **I** Cilia length distribution of POMC-eGFP neurons from mice fed chow or HFD for 16 weeks. Data are presented as mean ± SEM. Statistical differences were evaluated by using: **A** Two-way ANOVA followed by post hoc Sidak multiple comparison test; **B**, **D**, **H** unpaired two-tailed Student *t*-test; **C** two-way ANOVA, post hoc Holm-Sidak multiple comparison test. *p < 0.05, **p < 0.01, ***p < 0.001, ****p < 0.0001. *n* = 4/group.

Prior studies have shown that long-term consumption of a pro-obesogenic HFD reduces the length of primary cilia in the hypothalamus of mice [36]. Importantly, it was not determined in which type of neurons this effect was occurring. Here we assessed the effect of HFD specifically on POMC neurons, by using male POMC-eGFP mice fed with a chow or HFD for 16 weeks. As expected, HFD feeding significantly increased the body weight of mice (Fig. 1A, B), together with decreasing glucose tolerance in a glucose tolerance test (Fig. 1C, D). The percentage of POMC neurons with cilia was 76.38 ± 3.61%, in chow diet-fed mice, while this number was significantly reduced in POMC neurons of diet-induced obese mice (44.80 ± 5.35%) (Fig. 1E–H). Supplementary Figs. 1 and 2 describe the process of cilia selection, to determine that the cilia analyzed correspond to each POMC-eGFP cell. In addition, the z-plane in which each marked POMC-eGFP neuron was found was closely analyzed, to confirm which of the cilia identified matched the cell´s position (Supplementary videos S1 and S2). Cilia length, which on average was 7.00 ± 0.21 μm in cilia from POMC neurons of chow-fed animals and 6.56 ± 0.25 μm in cilia of POMC neurons from mice that consumed the HFD, was not affected by diet consumption (Supplementary Fig. 3A). Cilia volume, surface, and cilia bending (determined as the ratio of ciliary length and the length of the orientation vector) in POMC neurons were not affected by long-term HFD feeding (Supplementary Fig. 3). However, cilia length was significantly reduced in cilia of non-POMC cells, which considers all the other cilia identified in the tissue (the other types of neurons and astrocytes) (Supplementary Fig. 3A). Cilia volume, surface and cilia bending were significantly reduced in non-POMC cells of mice fed with the HFD (Supplementary Fig. 3B-D). Finally, we plotted the frequency of different cilia lengths in POMC neurons; we observe that this was not significantly affected by HFD consumption (Fig. 1I). These results indicate that consumption of HFD reduces the number of cilia, their volume, surface, and bending in hypothalamic neurons, with different effects depending on the type of neuron.

### PA reduces the percentage of ciliated cells and primary cilium length in hypothalamic neurons

**Fig. 2:**
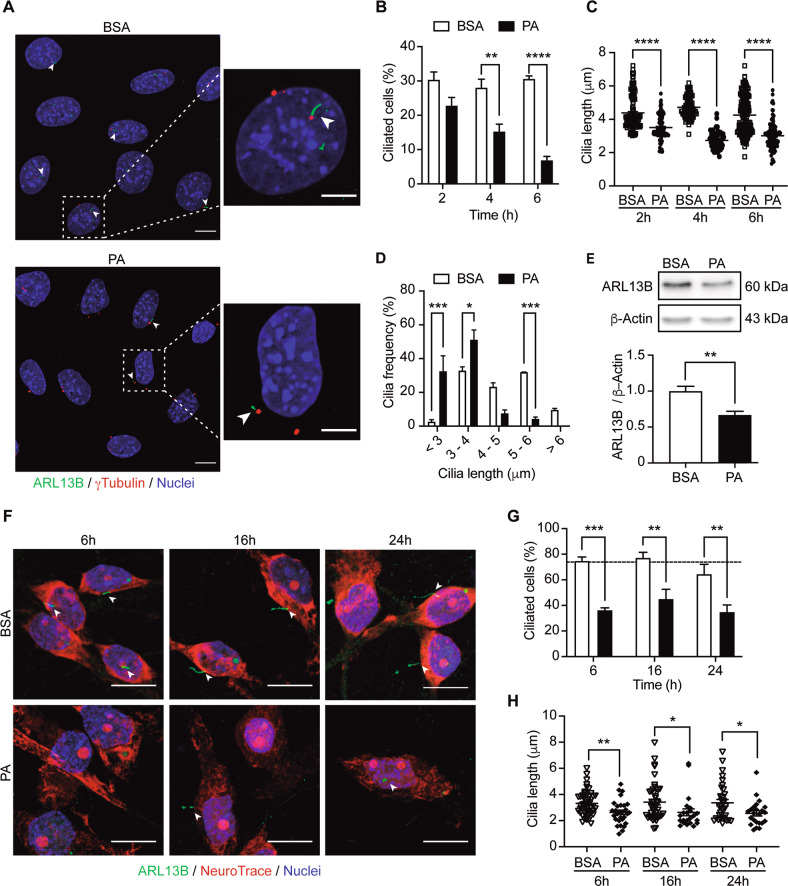
Palmitic acid reduces the percentage of ciliated cells and primary cilium length in hypothalamic neurons. **A** Representative confocal images of N43/5 hypothalamic neuronal cells treated with BSA (vehicle) or palmitic acid (PA, 100 μM) during 6 h, stained against ARL13B (ADP-ribosylation factor-like protein 13B, cilia axoneme) and γ-tubulin (cilia basal body). Scale bar: 10 µm. Inserts show a magnification of one cell within the dotted square. Insert scale bar: 5 µm. Arrowheads indicate the primary cilium. Quantification of **B** percentage of ciliated cells, **C** cilia length, and **D** cilia length distribution of N43/5 hypothalamic cells treated with BSA (vehicle) or palmitic acid (PA) during 2 h, 4 h, and 6 h and stained against ARL13B. **E** Representative western blot of ARL13B proteins levels in N43/5 cell lysates, incubated with BSA or PA (100 μM) for 6 h with the respective quantification. **F** Representative confocal images of primary hypothalamic neurons treated with BSA or PA (100 μM) for 6 h, 16 h, and 24 h, stained against ARL13B and with the neuronal marker NeuroTrace, with their respective quantifications (**G**, **H**). Arrowheads indicate the primary cilium. Scale bar: 10 µm. Nuclei were stained with Hoechst (blue). Data are presented as mean ± SEM. Statistical differences were evaluated by using: **B**, **D**, **G** Two-way ANOVA followed by post hoc Sidak multiple comparison test; **C** one-way ANOVA, post hoc Tukey multiple comparison test; **E**, **H** unpaired two-tailed Student *t*-test. *p < 0.05, **p < 0.01, ***p < 0.001, ****p < 0.0001. *n* = 3.

As shown in Fig. 1H and Supplementary Fig. 3A, and consistent with a previous study [36], HFD consumption affects the number of ciliated neurons and cilium length in the hypothalamus. Based on our previous studies, we know that chronic consumption of HFD changes the composition of fatty acids in the brain [7, 33]. We have focused our attention on palmitic acid (PA), the main SatFA in HFD, which, as demonstrated in previous work, accumulates in the hypothalamus of rodents following chronic HFD feeding [8]. Here we evaluated if exposure to this SatFA affects ciliogenesis in hypothalamic neurons. PA exposure over time decreased both the percentage of ciliated cells and cilia length in N43/5 cells, a model of hypothalamic POMC neurons [37], overtime (Fig. 2A–C). Our results show an increase in the frequency of shorter cilia (less than 4 μm) in PA-treated cells compared to control cells (BSA, used as vehicle) (Fig. 2D). This result was confirmed by using two different markers of primary cilia: acetylated ɑ-tubulin (Supplementary Fig. 4A), which stains the ciliary axoneme [38], and ARL13B (Fig. 2A–D), a regulatory GTPase highly enriched in cilia [38]. ARL13B protein levels significantly decreased following 6 h of PA exposure (Fig. 2E). Treatment with an additional SatFA, stearic acid, the second SatFAs increased in the hypothalamus of mice chronically exposed to the HFD [8], also decreased the percentage of ciliated cells (Supplementary Fig. 4B). Conversely, exposure to the polyunsaturated fatty acid (PUFA) ɑ-linolenic acid, did not affect ciliogenesis (Supplementary Fig. 4B). This effect was replicated in primary hypothalamic neurons (Fig. 2F–H), where the percentage of ciliated neurons and cilia length decreased over time, when neurons are exposed to PA. Conversely, treatment with PA did not affect the number of cilia in primary hypothalamic astrocytes, however, their length was reduced (Supplementary Fig. 4C, D). Together, these results indicate that the effect of fatty acids on primary cilia is dependent on the class of fatty acid; SatFAs are the ones that produce a decrease in the percentage of cilia and cilia length. In addition, the effect of PA on primary cilia is cell-type dependent, as the reduction in cilia has been identified only in hypothalamic neurons but not in hypothalamic astrocytes.

### Autophagy impairment decreases ciliogenesis in hypothalamic neurons

**Fig. 3:**
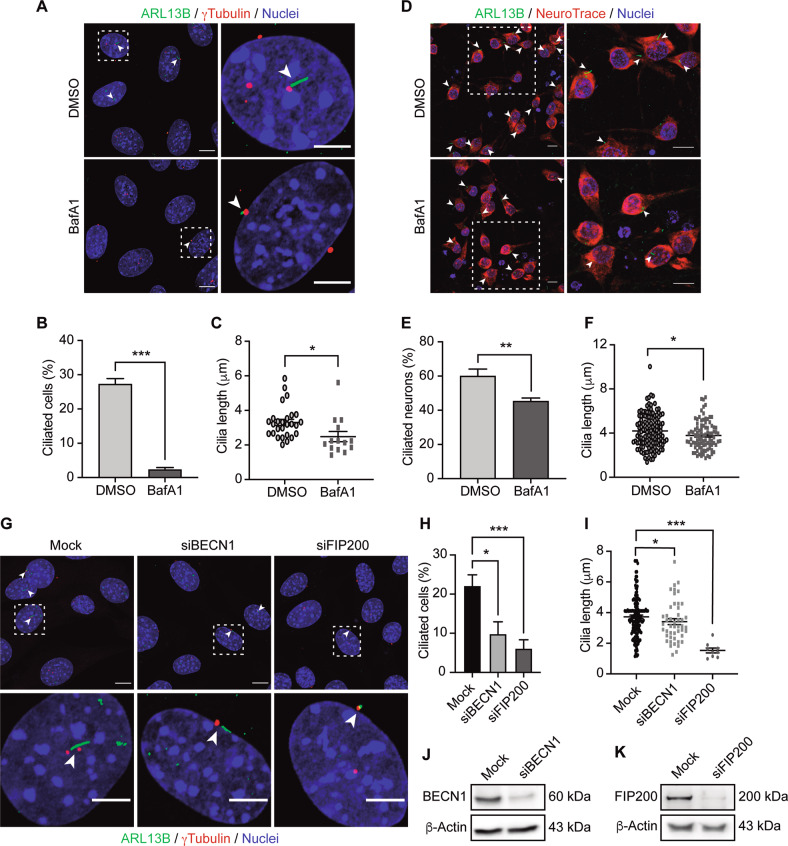
Autophagy impairment decreases ciliated cell percentage and primary cilium length in hypothalamic neurons. **A** Representative confocal images of N43/5 hypothalamic neuronal cells treated with the autophagic flux inhibitor BafA1 (100 nM) or its vehicle (DMSO) for 6 h and then stained against ARL13B (ADP-ribosylation factor-like protein 13B, cilia axoneme) and γ-tubulin (cilia basal body). Arrowheads indicate the primary cilium. Scale bar: 10 µm. Inserts show a magnification of one cell within the dotted square. Insert scale bar: 5 µm. Quantification of **B** percentage of ciliated cells and **C** cilia length of N43/5 hypothalamic cells treated as indicated in **A**. **D** Representative confocal images of primary hypothalamic neurons treated with DMSO or BafA1 (100 nM) during 6 h, stained against ARL13B and with the neuronal marker NeuroTrace, with their respective quantifications (**E**, **F**). Inserts show a magnification of cells within the dotted square. Arrowheads indicate the primary cilium. Scale bars: 10 µm. **G** Representative confocal images of N43/5 hypothalamic neuronal cells transfected with siRNA against BECN1 and FIP200. Scale bar: 10 µm. Inserts show a magnification of one cell within the dotted square. Insert scale bar: 5 µm. Arrowheads indicate the primary cilium. Nuclei were stained with Hoechst (blue). Quantification of **H** percentage of ciliated cells and **I** cilia length of N43/5 hypothalamic cells treated as indicated in **G**. Representative western blot showing protein levels of N43/5 cells depleted of **J** BECN1 and **K** FIP200. Cells were incubated with Lipofectamine RNAiMAX reagent only (Mock) as control. Data are shown as mean ± SEM. Comparisons between two conditions were made using the unpaired two-tailed Student *t*-test. One-way ANOVA was used for comparison of more than 2 groups, followed by Tukey’s post hoc adjustment. *p < 0.05, **p < 0.01, ***p < 0.001. *n* = 3.

Based on our previous findings, where we showed that PA impairs autophagy [31, 35], and consistent with previous work showing a specific interplay between autophagy and primary cilia [28, 39, 40], we hypothesize that the reduction in primary cilia number and length we observed in PA-treated hypothalamic neurons could be mediated by autophagy inhibition. Thus we exposed N43/5 cells (Fig. 3A–C) and primary hypothalamic neurons (Fig. 3D–F) to the classic autophagy flux inhibitor Bafilomycin A1 (BafA1) [41], and we determined that inhibition of the autophagic flux indeed decreased the percentage of ciliated cells and cilia length in both models. The same result was obtained when N43/5 cells were treated with chloroquine (CQ) (Supplementary Fig. 5A), an additional chemical inhibitor of the autophagic flux [41]. Importantly, even if BafA1 acutely increases [Ca]i (Supplementary Fig. 5B, C), this response is transient, as 6 h BafA1 pretreatment does not change the calcium influx induced by KCl, used as positive control to increase cytosolic calcium concentration (Supplementary Fig. 5D, E). To note, CQ does not modify [Ca]i (Supplementary Fig. 5B–E), suggesting the effect on cilia is directly caused by autophagy depletion and not related to calcium signaling, an additional element that has previously been shown to affect ciliogenesis [42]. Consistently, depletion of different essential autophagy proteins involved in the formation of the autophagosome, namely Beclin-1 (BECN1) and FIP200, significantly reduced the abundance of cilia and their length in N43/5 cells (Fig. 3G–K). Together these results indicate that inhibition of autophagy impairs ciliogenesis in hypothalamic neurons both at the step of autophagosome formation and during the later stages of the autophagic flux.

### Primary cilium dysfunction inhibits insulin signaling in hypothalamic neurons

Some types of ciliopathies such as Alström’s syndrome are characterized by early onset of type 2 diabetes, hyperinsulinemia, and obesity, suggesting a contribution of the primary cilium in its pathogenesis [43, 44].

**Fig. 4:**
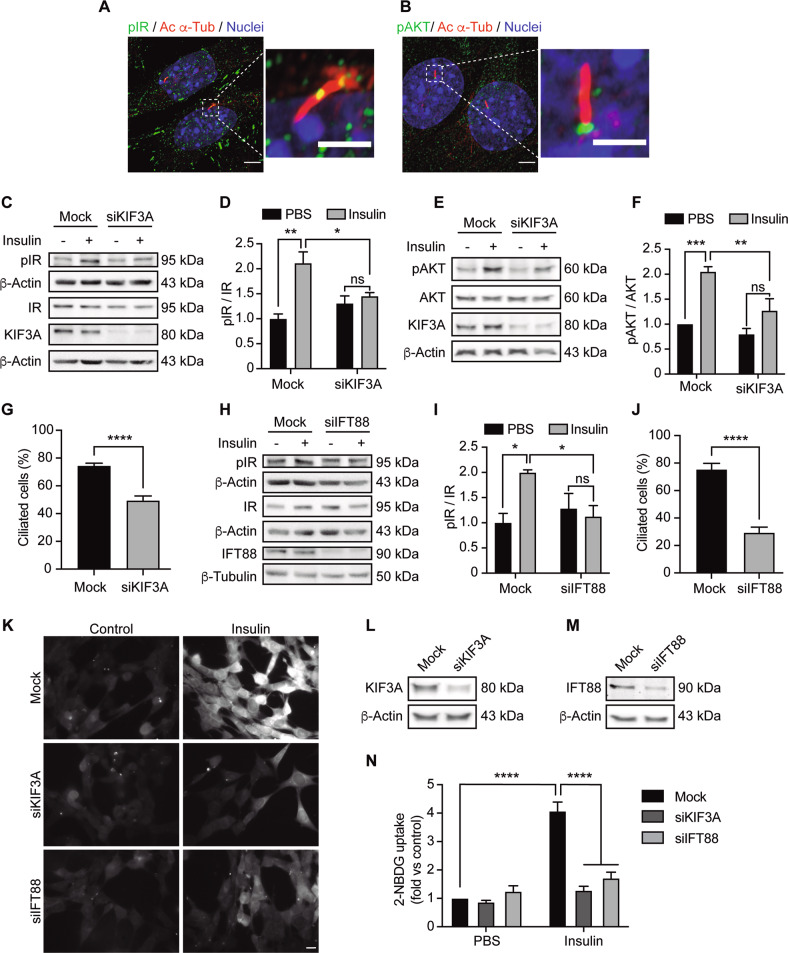
Primary cilium depletion reduced insulin signaling in hypothalamic cells. Representative confocal images showing **A** phospho-IR (pIR) and **B** phospho-AKT (pAKT) with primary cilium (acetylated α-tubulin, Ac α-tub) double immunostaining in N43/5 hypothalamic neuronal cells. Scale bar: 10 µm. Inserts show a magnification of one cilium within the dotted square. Insert scale bar: 2 µm. Nuclei were stained with Hoechst (blue). Representative blots of N43/5 hypothalamic cells transfected with siRNA against KIF3A followed by insulin (1 nM) or PBS (control) treatment for 3 min to evaluate IR phosphorylation (**C**) or 12 min to evaluate AKT phosphorylation (**E**), with their respective quantifications (**D**, **F**). **G** Percentage of ciliated cells in N43/5 hypothalamic cells depleted of KIF3A. **H** Representative blot of N43/5 hypothalamic cells transfected with siRNA against IFT88 followed by insulin (1 nM) or PBS (control) treatment for 3 min to evaluate IR phosphorylation, with their respective quantifications (**I**). **J** Percentage of ciliated cells in N43/5 hypothalamic cells transfected with siRNA against IFT88. **K** Representative images of 2-NBDG uptake in N43/5 hypothalamic cells transfected with siRNA against KIF3A and IFT88 and then stimulated with insulin 1 nM for 30 min, with its quantification (**N**). Scale bar: 10 μm. Representative western blots showing protein levels of N43/5 hypothalamic cells depleted of **L** KIF3A or **M** IFT88. As control condition, cells were incubated with Lipofectamine RNAiMAX reagent only (Mock). Data are presented as mean ± SEM. Comparisons between two conditions were made using the unpaired two-tailed Student *t*-test. Two-way ANOVA was used for comparison of more than 2 groups, followed by Sidak’s post hoc adjustment. *p < 0.05, **p < 0.01, ***p < 0.001, ****p < 0.0001. ns, not significant. *n* = 3.

To understand this potential relation, here we evaluated the role of the primary cilium in N43/5 cells in insulin signaling and glucose uptake (Fig. 4). First, we determined that, in addition to pancreatic β-cells [32], the active insulin receptor (IR) localizes at the primary cilium, as indicated by the presence of p-IR (Tyr1361) in the axoneme (Fig. 4A). Not only IR, but also additional proteins of the insulin receptor-dependent signaling localize at the primary cilium, such as the active form of protein kinase B, also known as AKT (Ser473), which has been identified at the ciliary base of N43/5 cells (Fig. 4B), suggesting that primary cilia sensitize insulin receptor signaling.

As shown in Fig. 2, PA exposure depletes primary cilia in hypothalamic neurons, and at the same time, blocks insulin-dependent signaling [31]. Thus, we evaluated whether depletion of primary cilia affects insulin sensitivity, as the IR localizes at the primary cilium. To this end, we determined the effect of the siRNA-dependent downregulation of essential genes for cilia assembly, *Kif3a* and *Ift88*, on insulin signaling and insulin-dependent glucose uptake. KIF3A knockdown, which reduced the percentage of ciliated cells (Fig. 4G), blunted insulin-dependent signaling and insulin-dependent glucose uptake, as indicated by the decrease in p-IR and p-AKT levels following insulin treatment (Fig. 4C–F) and by the reduction in insulin-dependent glucose uptake (Fig. 4K–N). In accordance with these results, downregulation of IFT88, which reduced the percentage of cilia (Fig. 4J), decreased the phosphorylation of IR (Tyr1361) induced by insulin treatment (Fig. 4H, I), and impaired insulin-dependent glucose uptake in N43/5 cells (Fig. 4K–N). These results confirm the primary cilium in hypothalamic neuronal cells is necessary to mediate insulin signaling.

### Induction of ciliogenesis stimulates insulin signaling

**Fig. 5:**
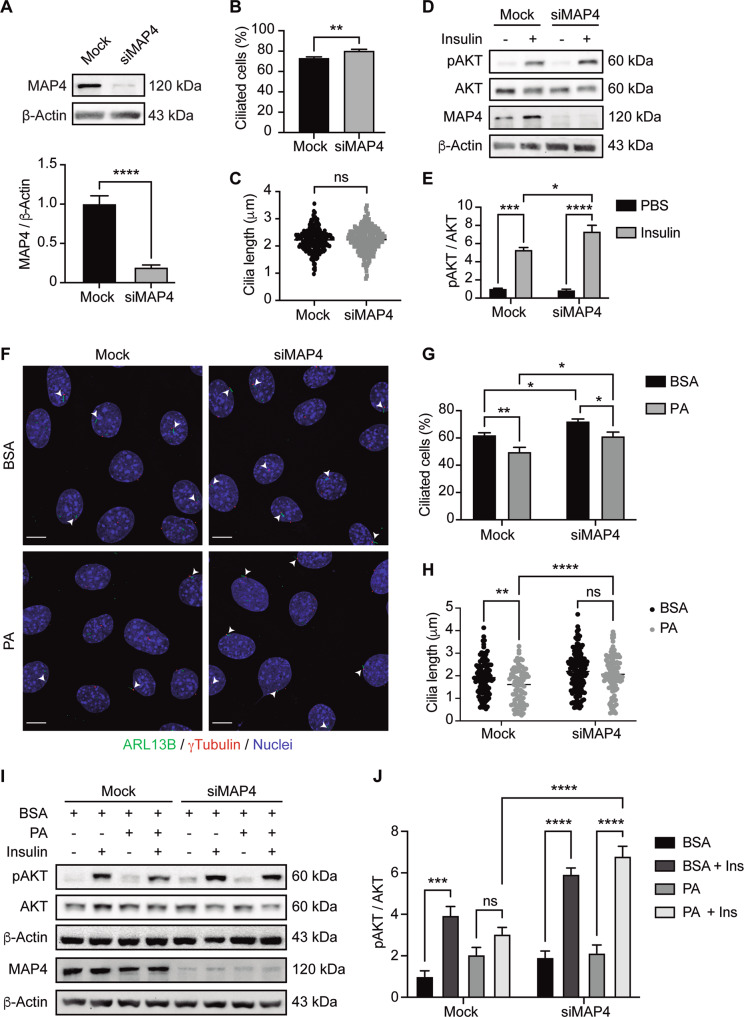
Increase in the percentage of ciliated cells promotes insulin signaling. **A** Representative blots of N43/5 cells transfected with siRNA against MAP4 showing MAP4 protein level in control condition (Mock) and following siRNA-mediated downregulation (siMAP4), and the respective quantification. β-actin was used as loading control. **B** Percentage of ciliated cells in N43/5 hypothalamic cells depleted of MAP4. **C** Cilia length of N43/5 cells in mock conditions or following MAP4 siRNA mediated downregulation. **D** Representative blots of N43/5 hypothalamic cells transfected with siRNA against MAP4 followed by insulin (1 nM) or PBS (control) treatment for 12 min to evaluate AKT phosphorylation (**D**), with their respective quantifications (**E**). **F** Representative confocal images of N43/5 cells transfected with siRNA against MAP4 and then exposed to BSA (vehicle) or 100 µM palmitic acid (PA) for 2 h. After fixation, cells were stained against ARL13B (ADP-ribosylation factor-like protein 13B, cilia axoneme) and γ-tubulin (cilia basal body). Arrowheads indicate the primary cilium. Scale bar: 10 µm. Quantification of the percentage of ciliated cells (**G**) and cilia length (**H**) of N43/5 cells exposed to the treatments indicated in **F**. **I** Representative blots of N43/5 cells transfected with siRNA against MAP4 followed by BSA or PA (100 µM) treatment for 2 h and then stimulated with insulin (1 nM) during 12 min, to evaluate AKT phosphorylation, with the respective quantification (**J**). Data are presented as mean ± SEM. Comparisons between two conditions were made using the unpaired two-tailed Student *t*-test. Two-way ANOVA was used for comparison of more than 2 groups, followed by Sidak’s post hoc adjustment. *p < 0.05, **p < 0.01, ***p < 0.001, ****p < 0.0001. ns, not significant. *n* = 3.

**Fig. 6:**
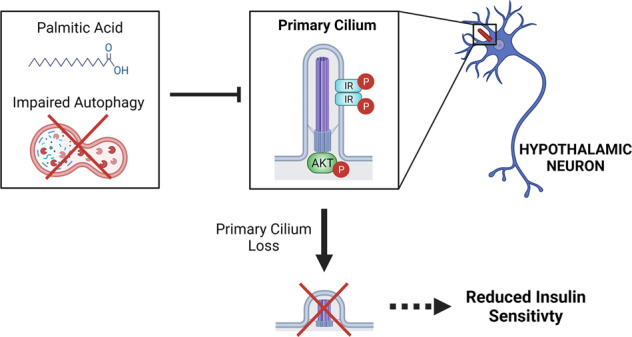
Primary cilium loss induced by palmitic acid or autophagy impairment reduces insulin sensitivity. Our results indicate that conditions which impair autophagy, such as PA exposure or chemical and genetic inhibition of autophagy, obtained by treatment with the autophagy inhibitor Bafilomycin A1 or Chloroquine or downregulation of essential autophagy genes, causes cilia loss in hypothalamic neuronal cells. We also show that proteins required for insulin signaling (IR and AKT) localize at the primary cilium, promoting insulin sensitivity. Consistently, conditions that cause cilia loss impair insulin signaling in hypothalamic neurons. p-IR: phosphorylated insulin receptor; p-AKT: phosphorylated protein kinase B (also known as AKT). Figure created with BioRender.com.

Next, we assessed if induction of ciliogenesis is sufficient to promote insulin signaling. We silenced in N43/5 cells the protein microtubule-associated protein 4 (MAP4), a protein of the MAP2/TAU family, which promotes the polymerization and stability of cytoplasmic microtubules (Fig. 5A) [45] and whose downregulation has previously been shown to increase the percentage of ciliated cells and cilia length in retinal pigmented epithelial cells [45]. Our results show that MAP4 silencing significantly increased the percentage of ciliated cells (Fig. 5B) without affecting cilia length (Fig. 5C). Then we evaluated the effect of insulin on its signaling pathway in cells in presence or absence of MAP4. Our results indicate that MAP4 downregulation, which increases the percentage of N43/5 ciliated cells, further enhances the phosphorylation of AKT compared to control, when cells are exposed to insulin (Fig. 5D, E). Altogether, these results show that ciliogenesis can indeed promote insulin signaling (Fig. 6).

In addition, we evaluated if induction of ciliogenesis prevents the effect of PA on insulin signaling, as acute exposure to PA reduces insulin signaling [31], as indicated by the levels of AKT phosphorylation. To this aim, we downregulated MAP4 in the presence or absence of PA and we determined the levels of AKT phosphorylation in response to insulin. Our data show that MAP4 silencing increases the number of primary cilia and cilia length, in PA-exposed cells (Fig. 5F–H) and restores insulin-mediated AKT phosphorylation, reduced by PA treatment (Fig. 5I, J). These results show that enhanced ciliogenesis prevents the effect of PA on insulin signaling.

## Discussion

The present study demonstrates that chronic consumption of an HFD in male mice reduces the number of cilia in POMC-expressing neurons (Fig. 1). Cilia length is also significantly reduced in non-POMC cells (other hypothalamic neurons and astrocytes) (Supplementary Fig. 3), possibly due to the accumulation of PA in the hypothalamus. We show that N43/5 cells and primary hypothalamic neurons exposed to a pro-obesogenic concentration of PA decreased their percentage of ciliated cells and cilia length (Fig. 2). The treatment of primary cultures of hypothalamic astrocytes with PA also diminished cilia length, but not the number of cilia (Supplementary Fig. 4). These results suggest cell specificity for the effect of the HFD-derived PA in the regulation of ciliogenesis (Fig. 6).

Chronic consumption of HFD is the main cause of visceral obesity, glucose intolerance, and insulin resistance [46]. Recently there has been increased attention towards the relation between obesity and primary cilia. Han et al. [36] demonstrated that long-term consumption of a pro-obesogenic HFD reduces cilia length in hypothalamic neurons of mice; however, they did not differentiate among types of hypothalamic neurons. Using a different cell model, Ritter et al. [47] showed that obese human donors exhibit shortened primary cilia and a reduced number of ciliated cells in adipose-derived mesenchymal stem cells (ASCs). However, the current study shows the effect of SatFAs on ciliogenesis and primary cilia length in neurons for the first time, specifically evaluating this effect on POMC neurons. We also demonstrated that the effect of fatty acids on ciliogenesis depends on the type of fatty acid; only SatFAs reduced the percentage of ciliated neurons and cilia length, at least in vitro.

In this work, we determined in vivo the chronic effect of HFD on the primary cilium, showing that long-term exposure depletes cilia in POMC neurons (Fig. 1). Conversely, in vitro, we showed that the effect of SatFAs on cilia is acute and maintained over time, at least up to 24 h, in primary hypothalamic neurons (Fig. 2). These results suggest that future experiments should be performed to determine if consumption of HFD has also a rapid effect on primary cilia in vivo. This might be of particular relevance at the level of arcuate neurons, which, due to their position next to the median eminence, are highly sensitive to changes in hormones and nutrients, such as fatty acids [48], finally affecting the activation of different cellular signaling pathways that depend on the primary cilium (i.e., leptin signaling or, as we showed, insulin signaling). It is tempting to speculate that the primary cilium, which is enriched in G-protein-coupled receptors [49], might contain fatty acid receptors such as GPR40 or GPR120, which are expressed at the level of the hypothalamus, rapidly activating cellular pathways involved in the control of energy homeostasis [50]. Furthermore, while the organism quickly responds to a short-term dietary-dependent fatty acid exposure by modulating intracellular pathways involved with lipid metabolism, this response is disrupted when the exposure is chronic, leading to the over accumulation of PA within the cell, promoting metabolic complications [51]. As such, short-time HFD effects might be even opposite on ciliogenesis, such as in the case of autophagy that, even if impaired in the hypothalamus after chronic HFD feeding, is enhanced in hypothalamic neurons of mice fed with an HFD for 1–3 days [52].

We recently showed that PA, in addition to its effect on the primary cilium, also impairs autophagy in hypothalamic neurons [31, 35]. Here we show that both pharmacological and genetic inhibition of autophagy decrease the percentage of ciliated cells and cilia length in neurons (Fig. 3), suggesting that PA impairs ciliogenesis in hypothalamic neurons through the inhibition of autophagy. An essential body of evidence shows that autophagy is one of the primary regulators of ciliogenesis in different cell types. Indeed, genetic [39, 40] and pharmacological [53, 54] manipulation of autophagy control ciliogenesis in both basal and nutrient-starvation conditions. Tang et al. [40] showed that autophagy promotes ciliogenesis through selective degradation of the Oral-facial-digital syndrome 1 (OFD1) protein localized at the centriolar satellites in retinal pigmented epithelial (RPE) cells and mouse embryonic fibroblasts (MEFs) [40, 55] reported that Peroxisome Proliferator Activated Receptor Alpha (PPARA), a nutrient-sensing receptor, induces ciliogenesis controlling the expression of autophagic genes in three different cell types (RPE, MEFs, and human kidney-2 (HK-2)) [55]. In addition, according to our data, it has been shown that autophagy inhibition decreases cilia length in HK-2 cells [55], and conversely, that autophagy activation promotes cilia elongation [40, 56]. In contrast with the findings mentioned above, other studies show that autophagy inhibition, using either 3-methyladenine or ATG5 KO cells, increases cilia length in MEFs [39, 57], suggesting that the role played by this lysosomal degradative pathway might depend on the cell type or be context-dependent, possibly to guarantee that the primary cilium is formed properly [58]. However, whether autophagy controls ciliogenesis and primary cilium length in hypothalamic neurons was previously unknown. Here we demonstrated that autophagy impairment decreases ciliogenesis and cilia length in primary cultures of hypothalamic neurons and in N43/5 cells (Fig. 3). We might speculate that autophagy inhibition prevents the degradation of selective regulators of ciliogenesis; thus influencing cilia formation, elongation or resorption. In this context, the decreased levels of ARL13B that we observed in hypothalamic N43/5 cells exposed to PA (Fig. 2E) could be the result or the cause of cilia resorption. Importantly, we showed that PA induces an increase in cytosolic calcium [31], which might affect cilia length, as previous work has demonstrated that the length of the primary cilium is negatively correlated with cytosolic calcium concentration, at least in mammalian epithelial and mesenchymal cells [59]. This suggests that not only autophagy inhibition, but also calcium fluxes induced by PA, might be affecting primary cilia in our model, an hypothesis that should be further investigated in the near future. Despite this, the leading role of autophagy in the regulation of primary cilia length is demonstrated by the action of chloroquine which inhibits autophagy and depletes primary cilia without increasing cytosolic calcium in N43/5 cells (Supplementary Fig. 5).

As mentioned before, primary cilia are sensory organelles that detect changes in the extracellular environment integrating and transmitting signaling information to the cell regulating cellular and physiological processes [60]. Depletion of primary cilia in the hypothalamus, as well as aberrant localization and signaling of ciliary receptors, causes obesity and impairs glucose homeostasis [21, 36, 61–66]. It has been demonstrated that stereotaxic injection of siRNAs against ciliary proteins in the mediobasal hypothalamus reduces the insulin response [36], and that the specific depletion of primary cilia in hypothalamic neurons is sufficient to increase plasma insulin levels in mice [61], suggesting that hypothalamic primary cilia might be involved in the regulation of insulin signaling and insulin plasma levels.

We showed that the insulin receptor localizes at the primary cilium in hypothalamic neurons (Fig. 4A). Additional proteins of the insulin receptor-signaling pathway localize at the primary cilium, such as the active form of AKT (p-AKT Ser473), which has been identified at the ciliary base (Fig. 4B), suggesting that primary cilia participate in the activation of insulin signaling in this cell type. It was previously shown that insulin receptors localize to the primary cilia of pancreatic β cells [32, 67], where primary cilia are necessary for proper insulin signaling. Two studies have dealt with this: Gerdes et al. [67] indicate that the active IR has to translocate to the cilium, following insulin stimulation, to activate the insulin-dependent signaling pathway. However, our results confirm the recent study of Li et al. [32], where authors showed that the IR constitutively localizes at the cilium, where it is stably active. It is currently unknown why the levels of phosphorylation of the IR at the cilium are higher, however, this might be associated, as the authors suggest, with the cilia high surface-to volume ratio, which enhances the possibility of interaction between the IR and its scaffold proteins. Indeed, for this reason, the primary cilium has been defined as a “signaling platform” [68]. Previous work reported that also the Insulin-like growth factor 1 receptor (IGF1R), localized at the cilium, is more sensitive to insulin stimulation than the receptors localized in the plasma membrane [69].

Finally, we also demonstrate how reducing the percentage of ciliated cells, using siRNAs against IFT88 and KIF3A impaired insulin-dependent signaling and glucose uptake in N43/5 cells (Fig. 4), and conversely that increasing the number of ciliated cells increase insulin signaling (Fig. 5), supporting the idea that primary cilia are essential to mediate insulin response in hypothalamic neuronal cells. The expression of the insulin-sensitive glucose transporter, GLUT4, has been demonstrated in the hypothalamus of rodents, mostly in neurons [70], where it is involved in hypothalamic regulation of food intake, energy expenditure, and whole-body glucose homeostasis [71, 72]. The selective knockout of brain GLUT4 in mice produces glucose intolerance, hepatic insulin resistance, and reduces glucose uptake in the brain, indicating a critical role for brain GLUT4 in sensing and responding to changes in blood glucose [72]. Although so far no studies have reported the presence of GLUT4 at the primary cilium, it has been shown that RAB10, which is required for GLUT4 translocation from intracellular storage to the plasma membrane [73], localizes at the base of the cilia of renal epithelial cells [74]. Future studies need to determine whether the decrease in insulin-dependent glucose uptake in conditions of reduced ciliogenesis is caused by a decrease in the presence of GLUT4 at the primary cilium.

Our present findings demonstrate a role for SatFAs in the control of ciliogenesis and cilia length in hypothalamic neurons, indicating an autophagy-dependent mechanism. Our results also show the cilium-related regulation of insulin signaling in hypothalamic neurons, offering a potential new therapeutic avenue for obesity and insulin resistance.

## Materials and methods

### Animals and body weight measurements

All animal protocols in this study were approved by the Pontificia Universidad Católica de Chile Animal Ethics Committee (protocols 190606002, 170706033). The number of animals for each experiment was calculated to be a low as possible, yet enabling us to gain meaningful/reliable data, according to the work we previously published [34], as calculated by the free online sample size calculator at http://www.biomath.info. Animals were randomly assigned to the 2 test groups and always maintained in the same environment to avoid bias. All measurements and tests were performed in a blinded fashion. Eight-week-old C57BL/6J-Tg(Pomc-eGFP)1Low/J (Jackson Laboratory, #009593, Bar Harbor, ME, USA) male mice were fed with a regular diet (0001495, Prolab® RMH 3000, LabDiet, St. Louis, MO, USA) or a 40% high-fat diet (HFD, D12079B, Research Diets, New Brunswick, NJ, USA) for 16 weeks. Mouse body weight was measured weekly.

### Glucose tolerance test

Six-hour-fasted mice were injected intraperitoneally with glucose (2 g/kg of body weight) to perform the glucose tolerance test. Glucose concentrations were measured before and 15, 30, 45, 60, 90, and 120 min after the challenge in an Accu-Chek® Performa glucometer (Roche Diagnostics, S.L., Spain). Immediately after the end of the experiment, mice were placed in a new clean cage with food and water. These procedures were performed as suggested by the NIH Mouse Metabolic Phenotyping Center (MMPC) Consortium [75].

### Immunofluorescence and confocal microscopy

For staining neuron cilia in mice, animals under anesthesia (Ketamine 80 mg/kg and Xylazine 12 mg/kg) were perfused transcardially with 4% PFA, and brains were post-fixed in 4% PFA for 24 h and infiltrated with 20–30% sucrose. Brain sections of 30 µm thickness were made using a cryostat (Leica Biosystems, IL, USA) at −20 °C. Fixed brain sections were blocked with 3% bovine serum albumin (BSA, Winkler, Chile) in phosphate-buffered saline (PBS) and permeabilized with 0.25% Triton X-100 for 1 h at room temperature. Then, brain sections were incubated overnight at 4 °C with the primary antibody against adenylate cyclase 3 (AC3; 1:300, PA5-35382, Invitrogen, Carlsbad, CA, USA) prepared in 0.05% Triton X-100 and 3% BSA in PBS, followed by conjugation with its secondary antibody (Alexa Fluor, Life Technologies, Carlsbad, CA, USA), for 1 h at room temperature.

N43/5 cells and primary cultures of hypothalamic neurons were treated as indicated in each experiment, then both the percentage of ciliated cells and primary cilium length were evaluated by immunofluorescence (IF). For cilia analysis in N43/5 cells, cells were fixed with 4% PFA for 20 min at room temperature, blocked in 3% BSA in PBS for 1 h, and stained with primary antibodies against ADP-ribosylation factor-like protein 13B (ARL13B; 1:200; 17711-1-AP, Proteintech, Rosemont, IL, USA) or acetylated α-tubulin (1:300; T6793, Sigma-Aldrich, St. Louis, MO, USA) at 4 °C overnight. To stain the basal body of primary cilia, cells were incubated with anti–γ-tubulin antibody (1:300; T66557, Sigma-Aldrich) at 4 °C overnight. To evaluate the presence of insulin-related signaling proteins at the primary cilium cells were stained with primary antibodies against phospho-Insulin Receptor (phospho Tyr1361; 1:200; ab60946, Abcam, UK) and phospho-AKT (Ser473; 9271, Cell Signaling Technology, Danvers, Massachusetts, USA), at 4 °C overnight, followed by conjugation with its secondary antibody (Alexa Fluor, Life Technologies), for 1 h at room temperature. Nuclei were counterstained with Hoechst 33342 (10 mg/ml) (Molecular Probes, Eugene, OR, USA). Confocal fluorescence microscopy assessments were performed using an LSM 880 Zeiss inverted confocal microscope with Airyscan detection and Nikon Ti-E C2si microscope (Unidad de Microscopía Avanzada UC (UMA UC); Oberkochen, Germany). Contrast and/or brightness adjustment and cropping of final images were done using ImageJ, with identical settings applied to all images from the same experiment.

### Primary cilium analysis in tissues

Images were obtained using a Zeiss LSM 880 inverted confocal microscope with Airyscan detection (UMA UC), using a 40x objective with 1.2 NA (pixel size 0.08 µm). A total of 15 z-stack (0.14 µm/slice) images were taken for each treatment (Chow and HFD) in 3 different channels: primary cilia (568 nm), POMC-eGFP cells (488 nm), and cell nuclei (405 nm). Image automated and curated segmentation of cell and primary cilia were performed using Morphometry software written in IDL (ITT Visual Information Solutions), freely available in the GitHub repository (https://github.com/KanchanawongLab/Morphometry). Initial image thresholding of all images was followed by generation of binary masks for primary cilia, indicated as regions of interest (ROIs), area of POMC-eGFP cells, and total cell area. Primary cilia associated with POMC cells were determined by superposition of the corresponding binary masks, and ROIs within each cell’s area were selected for further analysis. For the selection of primary cilia in the z-stack images, the binary masks were used for selection of the ROIs (Supplementary Figs. 1 and 2 and Supplementary Videos 1 and 2). Quantification of 3D cilia length, volume, surface, and cilia bending index were performed using ImageJ’s plugin CiliaQ (CiliaQ-0.1.4, CiliaQ, Editor_JNH-0.0.3, and CiliaQ Preparator_JNH-0.1.1). All signals that intersected with the limit of any axis were excluded as well as all measured signals that had a length less than 0.68 µm or a volume <0.6 µm^3^.

### Primary cilium analysis in cells

The percentage of cells with primary cilia was evaluated by counting the number of ciliated cells versus their total number. Approximately 100–150 cells per sample from three different experiments were analyzed; each sample was visualized in duplicate. Cilia images were taken by z-stacking using a confocal microscope, reconstructed in 3D projections, and analyzed with ImageJ to determine primary cilium length as described by Dummer et al. [76].

### Primary culture of hypothalamic neurons

Primary cultures of hypothalamic neurons were prepared from E18 embryos from pregnant Sprague-Dawley rats. Embryos were euthanized by decapitation, and the hypothalami were dissected free of extraembryonic membranes using a magnifying glass. Then the hypothalami were digested using trypsin-EDTA in HBSS (10 min, 37 °C). Tissues were washed and dissociated using high-glucose Dulbecco’s modified Eagle’s medium (HG-DMEM; D2902, Sigma-Aldrich) supplemented with horse serum (adhesion medium) by pipetting with a Pasteur pipet. The resulting cell suspension was sifted using two cell strainers (70 and 40 μm). Then the cells were seeded on poly-L-lysine-covered vessels. After a 2-h incubation in the adhesion medium, the media was changed to neurobasal medium (21103049, Gibco, USA) supplemented with B27 (17504044, Gibco), L-glutamine (SH30034.01, Hyclone, Logan, Utah, USA) and penicillin/streptomycin (15140122, Gibco). Half of the medium was changed after three days, followed by a complete medium change every 3 days until day in vitro (DIV) 10. From DIV2, 3 µM AraC (C6645, Sigma-Aldrich) was added to cultured hypothalamic cells, to inhibit uncontrolled proliferation of non-neuronal cells [77].

### Cell line culture and treatments

N43/5 cells (Cellutions Biosystems, Canada) were cultured in high glucose Dulbecco’s modified Eagle medium (HG-DMEM) (11995-040, Gibco) supplemented with 10% fetal bovine serum (FBS) (10437028, Gibco), 100 U/ml penicillin-streptomycin (15140122, Gibco) and maintained at 37 °C with 5% CO_2_. Cells were routinely tested using EZ-PCR™ Mycoplasma Detection Kit (20-700-20, Biological Industries, Israel). Changes in the percentage of ciliated cells and in cilia length after exposure to different fatty acids were evaluated. To this end, cells were incubated with HG-DMEM supplemented with 2% FBS 24 h before treatments. Then cells were exposed to 100 μM PA (P0500, Sigma-Aldrich), 100 μM SA (stearic acid) or 100 μM ALA (α-linolenic acid) (Sigma-Aldrich) conjugated to fatty acid-free bovine serum albumin (BSA) (152401, MP Biomedicals, Santa Ana, CA, USA). The concentration of PA and SA has been determined based on a lipidomic analysis that assessed the amount of different fatty acids in the brain of male mice following feeding with and HFD for 16 weeks [7, 78], as such it mimics the amount of PA or SA in the brain of a long-term diet-induced obese mouse. BSA treatment was used as control.

In order to assess the changes in primary cilia after the inhibition of autophagy, cells were treated with Bafilomycin A1 (BafA1; B1793, Sigma-Aldrich) or CQ (C6628, Sigma-Aldrich) at concentrations of 100 nM and 30 μM, respectively. DMSO (BM-0660, Winkler) and PBS were used as controls for BafA1 and CQ, respectively.

To determine the effect of the knockdown of ciliary genes on insulin signaling in N43/5 cells, cells were silenced and serum-starved overnight in DMEM/F-12 medium (11330-32, Gibco), 16 h prior to treatments. Then, cells were co-treated with insulin (1 nM, Humulin R, human insulin, Lilly and Company, USA) for 3 min to evaluate IR phosphorylation or for 12 min to evaluate AKT phosphorylation [31]. PBS was used as control.

### siRNA transfection

Cells were cultured in six-well plates and transfected at 50% confluence with siRNAs targeting murine Beclin 1 (*Becn1*) (SASI_Mm01_00048143, Sigma-Aldrich), murine FAK family kinase-interacting protein of 200 kD (*Fip200*) (SASI_Mm01_00196359, Sigma-Aldrich), murine kinesin family member 3A (*Kif3a*) (SASI_Mm01_00024254, Sigma-Aldrich), murine intraflagellar transport protein 88 homolog (*Ift88*) (SASI_Mm01_00151435, Sigma-Aldrich), or murine microtubule associated protein 4 (*Map4*) (SASI_Mm01_00207888). Transfection was performed using Lipofectamine RNAiMAX Transfection Reagent (Invitrogen), according to the manufacturer’s instructions. Control cells were incubated with Lipofectamine RNAiMAX Transfection Reagent only. 48 h after siRNA transfection, cells were treated as indicated or directly lysed for protein or RNA extraction.

### Western Blot

Cells were lysed in RIPA buffer, and 30–40 μg of denatured proteins from each sample were resolved in 10% SDS-PAGE. Gels were transferred to nitrocellulose membranes and incubated with 5% BSA (BM-0150, Winkler)-tris buffered saline-0.1% Tween-20 (TBS-T) to block nonspecific binding. Membranes were incubated with the primary antibodies against ARL13B (17711-1-AP, Proteintech, IL, USA), BECN1 (H-300; sc-11427, Santa Cruz Biotechnology, Inc., Dallas, Texas, USA), FIP200 (17250-1-AP, Proteintech), anti-p-AKT (Ser473) (9271, Cell Signaling Technology), AKT (9272, Cell Signaling Technology), pIR (phospho Tyr1361; ab60946, Abcam), IR (sc-81465, Santa Cruz Biotechnology, Inc.), KIF3A (K3513, Sigma-Aldrich), IFT88 (13967-1-AP, Proteintech), and MAP4 (SAB1402840, Sigma-Aldrich), at dilution of 1:1000 in 5% BSA-TBS-T overnight on a rocking platform at 4 °C. Then, membranes were washed 3 times in TBS-T and revealed with the appropriate horseradish peroxidase-labeled secondary antibody (Goat Anti-Mouse IgG (H + L)-HRP Conjugate, 1706516; Goat Anti-Rabbit IgG (H + L)-HRP Conjugate, 1706515; Bio-Rad, CA, USA) and the chemiluminescent substrate. β-actin (1:10,000; A1978, Sigma-Aldrich) and β-tubulin (1:500; sc-5274, Santa Cruz Biotechnology, Inc.) were used as loading controls.

### 2-NBDG Uptake

To assess the insulin-dependent glucose uptake in N43/5 cells, cells were stimulated with insulin 1 nM for 30 min and incubated with the fluorescent analog of glucose 2-(N-(7-nitrobenz-2-oxa-1,3-diazol-4-il) amino)-2-deoxyglucose (2-NBDG, 300 mM) for 15 min at 37 °C as previously described [79]. Cells were transferred to an inverted Nikon Ti Eclipse microscope equipped with 40x oil objective [numerical aperture, N.A. 1.3]. A xenon lamp was coupled to the monochromator device (Cairn Research Ltd, Faversham, UK). Digital images were acquired by means of a cooled CCD camera (Hamamatsu ORCA 03, Japan). Images were quantified by ImageJ software (NIH, Bethesda, MD, USA).

### Statistical analysis

Results obtained are shown as a mean ± SEM for at least 3 independent experiments. Statistical analyses were performed with GraphPad Prism software (GraphPad Software Inc., San Diego, California, USA). Comparisons between two conditions were made using the unpaired two-tailed Student *t*-test. One- or two-way ANOVA was used for comparison of more than 2 groups as appropriate, followed by post hoc adjustment. *P* < 0.05 is considered to be statistically significant.

### Reporting summary

Further information on research design is available in the Nature Research Reporting Summary linked to this article.

## Supplementary information

Supplementary Information (Figures and experimental procedures)

Supplementary Video 1

Supplementary Video 2

Supplementary Material - Western Blots

Reporting Summary Aj-checklist

## Data Availability

Data included in the paper are available from the corresponding author upon request.

## References

[1] Ng M, Fleming T, Robinson M, Thomson B, Graetz N, Margono C (2014). Global, regional, and national prevalence of overweight and obesity in children and adults during 1980–2013: a systematic analysis for the Global Burden of Disease Study 2013. Lancet.

[2] Arner P, Ryden M (2015). Fatty acids, obesity and insulin resistance. Obes Facts.

[3] Frohnert BI, Jacobs DR, Steinberger J, Moran A, Steffen LM, Sinaiko AR (2013). Relation between serum free fatty acids and adiposity, insulin resistance, and cardiovascular risk factors from adolescence to adulthood. Diabetes.

[4] Chen W, Balland E, Cowley MA (2017). Hypothalamic insulin resistance in obesity: effects on glucose homeostasis. Neuroendocrinology.

[5] Kleinridders A, Ferris HA, Cai W, Kahn CR (2014). Insulin action in brain regulates systemic metabolism and brain function. Diabetes.

[6] Kang M, Lee A, Yoo HJ, Kim M, Kim M, Shin DY (2017). Association between increased visceral fat area and alterations in plasma fatty acid profile in overweight subjects: a cross-sectional study. Lipids Health Dis.

[7] Morselli E, Fuente-Martin E, Finan B, Kim M, Frank A, Garcia-Caceres C (2014). Hypothalamic PGC-1alpha protects against high-fat diet exposure by regulating ERalpha. Cell Rep.

[8] Vagena E, Ryu JK, Baeza-Raja B, Walsh NM, Syme C, Day JP (2019). A high-fat diet promotes depression-like behavior in mice by suppressing hypothalamic PKA signaling. Transl Psychiatry.

[9] Sterpka A, Chen X (2018). Neuronal and astrocytic primary cilia in the mature brain. Pharm Res.

[10] Sipos E, Komoly S, Acs P (2018). Quantitative comparison of primary cilia marker expression and length in the mouse brain. J Mol Neurosci.

[11] Satir P, Pedersen LB, Christensen ST (2010). The primary cilium at a glance. J Cell Sci.

[12] Haycraft CJ, Zhang Q, Song B, Jackson WS, Detloff PJ, Serra R (2007). Intraflagellar transport is essential for endochondral bone formation. Development.

[13] Pedersen LB, Rosenbaum JL (2008). Intraflagellar transport (IFT) role in ciliary assembly, resorption and signalling. Curr Top Dev Biol.

[14] Mariman EC, Vink RG, Roumans NJ, Bouwman FG, Stumpel CT, Aller EE (2016). The cilium: a cellular antenna with an influence on obesity risk. Br J Nutr.

[15] Lin F, Hiesberger T, Cordes K, Sinclair AM, Goldstein LS, Somlo S (2003). Kidney-specific inactivation of the KIF3A subunit of kinesin-II inhibits renal ciliogenesis and produces polycystic kidney disease. Proc Natl Acad Sci USA.

[16] Wheway G, Nazlamova L, Hancock JT (2018). Signaling through the primary cilium. Front Cell Dev Biol.

[17] Anvarian Z, Mykytyn K, Mukhopadhyay S, Pedersen LB, Christensen ST (2019). Cellular signalling by primary cilia in development, organ function and disease. Nat Rev Nephrol.

[18] Vaisse C, Reiter JF, Berbari NF. Cilia and obesity. Cold Spring Harb Perspect Biol. 2017;9:a028217. 10.1101/cshperspect.a028217 PMC5495057 28096262

[19] Hearn T. ALMS1 and Alstrom syndrome: a recessive form of metabolic, neurosensory and cardiac deficits. J Mol Med. 2019;97:1–17. 10.1007/s00109-018-1714-x PMC6327082 30421101

[20] Tsang SH, Aycinena ARP, Sharma T (2018). Ciliopathy: Bardet-Biedl syndrome. Adv Exp Med Biol.

[21] Lee CH, Song DK, Park CB, Choi J, Kang GM, Shin SH (2020). Primary cilia mediate early life programming of adiposity through lysosomal regulation in the developing mouse hypothalamus. Nat Commun.

[22] Coupe B, Ishii Y, Dietrich MO, Komatsu M, Horvath TL, Bouret SG (2012). Loss of autophagy in pro-opiomelanocortin neurons perturbs axon growth and causes metabolic dysregulation. Cell Metab.

[23] Malhotra R, Warne JP, Salas E, Xu AW, Debnath J (2015). Loss of Atg12, but not Atg5, in pro-opiomelanocortin neurons exacerbates diet-induced obesity. Autophagy.

[24] Kaushik S, Arias E, Kwon H, Lopez NM, Athonvarangkul D, Sahu S (2012). Loss of autophagy in hypothalamic POMC neurons impairs lipolysis. EMBO Rep.

[25] Quan W, Kim HK, Moon EY, Kim SS, Choi CS, Komatsu M (2012). Role of hypothalamic proopiomelanocortin neuron autophagy in the control of appetite and leptin response. Endocrinology.

[26] Avalos Y, Pena-Oyarzun D, Budini M, Morselli E, Criollo A (2017). New roles of the primary cilium in autophagy. Biomed Res Int.

[27] Shin JH, Bae DJ, Kim ES, Kim HB, Park SJ, Jo YK (2015). Autophagy regulates formation of primary cilia in mefloquine-treated cells. Biomol Ther.

[28] Yamamoto Y, Mizushima N (2021). Autophagy and ciliogenesis. JMA J.

[29] Yamamoto S, Kuramoto K, Wang N, Situ X, Priyadarshini M, Zhang W (2018). Autophagy differentially regulates insulin production and insulin sensitivity. Cell Rep.

[30] Kuramoto K, Kim YJ, Hong JH, He C (2021). The autophagy protein Becn1 improves insulin sensitivity by promoting adiponectin secretion via exocyst binding. Cell Rep.

[31] Hernandez-Caceres MP, Toledo-Valenzuela L, Diaz-Castro F, Avalos Y, Burgos P, Narro C (2019). Palmitic acid reduces the autophagic flux and insulin sensitivity through the activation of the free fatty acid receptor 1 (FFAR1) in the hypothalamic neuronal cell line N43/5. Front Endocrinol.

[32] Li Y, Shrestha PK, Wu YI. Primary cilia sensitize insulin receptor-mediated negative feedback in pancreatic β cells. bioRXiv:408914v1 [Preprint]. 2020. Available from: 10.1101/2020.12.02.408914.

[33] Morselli E, Frank AP, Palmer BF, Rodriguez-Navas C, Criollo A, Clegg DJ (2016). A sexually dimorphic hypothalamic response to chronic high-fat diet consumption. Int J Obes (Lond).

[34] Morselli E, Criollo A, Rodriguez-Navas C, Clegg DJ (2014). Chronic high fat diet consumption impairs metabolic health of male mice. Inflamm Cell Signal.

[35] Hernandez-Caceres MP, Cereceda K, Hernandez S, Li Y, Narro C, Rivera P (2020). Palmitic acid reduces the autophagic flux in hypothalamic neurons by impairing autophagosome-lysosome fusion and endolysosomal dynamics. Mol Cell Oncol.

[36] Han YM, Kang GM, Byun K, Ko HW, Kim J, Shin MS (2014). Leptin-promoted cilia assembly is critical for normal energy balance. J Clin Invest.

[37] Oh TS, Cho H, Cho JH, Yu SW, Kim EK (2016). Hypothalamic AMPK-induced autophagy increases food intake by regulating NPY and POMC expression. Autophagy.

[38] Tripathi P, Zhu Z, Qin H, Elsherbini A, Crivelli SM, Roush E (2021). Palmitoylation of acetylated tubulin and association with ceramide-rich platforms is critical for ciliogenesis. J Lipid Res.

[39] Pampliega O, Orhon I, Patel B, Sridhar S, Diaz-Carretero A, Beau I (2013). Functional interaction between autophagy and ciliogenesis. Nature.

[40] Tang Z, Lin MG, Stowe TR, Chen S, Zhu M, Stearns T (2013). Autophagy promotes primary ciliogenesis by removing OFD1 from centriolar satellites. Nature.

[41] Klionsky DJ, Abdel-Aziz AK, Abdelfatah S, Abdellatif M, Abdoli A, Abel S (2021). Guidelines for the use and interpretation of assays for monitoring autophagy. Autophagy.

[42] Zhdanov AV, Dmitriev RI, Papkovsky DB (2011). Bafilomycin A1 activates respiration of neuronal cells via uncoupling associated with flickering depolarization of mitochondria. Cell Mol Life Sci.

[43] Dassie F, Favaretto F, Bettini S, Parolin M, Valenti M, Reschke F (2021). Alstrom syndrome: an ultra-rare monogenic disorder as a model for insulin resistance, type 2 diabetes mellitus and obesity. Endocrine.

[44] Volta F, Gerdes JM (2017). The role of primary cilia in obesity and diabetes. Ann N. Y Acad Sci.

[45] Ghossoub R, Hu Q, Failler M, Rouyez MC, Spitzbarth B, Mostowy S (2013). Septins 2, 7 and 9 and MAP4 colocalize along the axoneme in the primary cilium and control ciliary length. J Cell Sci.

[46] Tchernof A, Despres JP (2013). Pathophysiology of human visceral obesity: an update. Physiol Rev.

[47] Ritter A, Friemel A, Kreis NN, Hoock SC, Roth S, Kielland-Kaisen U (2018). Primary cilia are dysfunctional in obese adipose-derived mesenchymal stem cells. Stem Cell Rep.

[48] Haddad-Tovolli R, Dragano NRV, Ramalho AFS, Velloso LA (2017). Development and function of the blood-brain barrier in the context of metabolic control. Front Neurosci.

[49] Mykytyn K, Askwith C. G-Protein-coupled receptor signaling in cilia. Cold Spring Harb Perspect Biol. 2017;9:a028183. 10.1101/cshperspect.a028183 PMC5585845 28159877

[50] Dragano NRV, Solon C, Ramalho AF, de Moura RF, Razolli DS, Christiansen E (2017). Polyunsaturated fatty acid receptors, GPR40 and GPR120, are expressed in the hypothalamus and control energy homeostasis and inflammation. J Neuroinflammation.

[51] Carta G, Murru E, Banni S, Manca C (2017). Palmitic acid: physiological role, metabolism and nutritional implications. Front Physiol.

[52] Reginato A, Siqueira BP, Miyamoto JE, Portovedo M, Costa SO, de Fante T (2020). Acute effects of fatty acids on autophagy in NPY neurones. J Neuroendocrinol.

[53] Kim ES, Shin JH, Park SJ, Jo YK, Kim JS, Kang IH (2015). Inhibition of autophagy suppresses sertraline-mediated primary ciliogenesis in retinal pigment epithelium cells. PLoS ONE.

[54] Bao Z, Huang W (2017). Thioridazine promotes primary ciliogenesis in lung cancer cells through enhancing cell autophagy. Int J Clin Exp Med.

[55] Liu ZQ, Lee JN, Son M, Lim JY, Dutta RK, Maharjan Y (2018). Ciliogenesis is reciprocally regulated by PPARA and NR1H4/FXR through controlling autophagy in vitro and in vivo. Autophagy.

[56] Wang S, Livingston MJ, Su Y, Dong Z (2015). Reciprocal regulation of cilia and autophagy via the MTOR and proteasome pathways. Autophagy.

[57] Struchtrup A, Wiegering A, Stork B, Ruther U, Gerhardt C (2018). The ciliary protein RPGRIP1L governs autophagy independently of its proteasome-regulating function at the ciliary base in mouse embryonic fibroblasts. Autophagy.

[58] Morleo M, Franco B. The autophagy-cilia axis: an intricate relationship. Cells 2019;8:905. 10.3390/cells8080905 PMC6721705 31443299

[59] Besschetnova TY, Kolpakova-Hart E, Guan Y, Zhou J, Olsen BR, Shah JV (2010). Identification of signaling pathways regulating primary cilium length and flow-mediated adaptation. Curr Biol.

[60] Christensen ST, Morthorst SK, Mogensen JB, Pedersen LB. Primary cilia and coordination of receptor tyrosine kinase (RTK) and transforming growth factor beta (TGF-beta) signaling. Cold Spring Harb Perspect Biol. 2017;9:a028167. 10.1101/cshperspect.a028167 PMC5453389 27638178

[61] Davenport JR, Watts AJ, Roper VC, Croyle MJ, van Groen T, Wyss JM (2007). Disruption of intraflagellar transport in adult mice leads to obesity and slow-onset cystic kidney disease. Curr Biol.

[62] Berbari NF, Lewis JS, Bishop GA, Askwith CC, Mykytyn K (2008). Bardet-Biedl syndrome proteins are required for the localization of G protein-coupled receptors to primary cilia. Proc Natl Acad Sci USA.

[63] Rahmouni K, Fath MA, Seo S, Thedens DR, Berry CJ, Weiss R (2008). Leptin resistance contributes to obesity and hypertension in mouse models of Bardet-Biedl syndrome. J Clin Invest.

[64] Wang Z, Li V, Chan GC, Phan T, Nudelman AS, Xia Z (2009). Adult type 3 adenylyl cyclase-deficient mice are obese. PLoS One.

[65] Guo DF, Lin Z, Wu Y, Searby C, Thedens DR, Richerson GB (2019). The BBSome in POMC and AgRP neurons is necessary for body weight regulation and sorting of metabolic receptors. Diabetes.

[66] Siljee JE, Wang Y, Bernard AA, Ersoy BA, Zhang S, Marley A (2018). Subcellular localization of MC4R with ADCY3 at neuronal primary cilia underlies a common pathway for genetic predisposition to obesity. Nat Genet.

[67] Gerdes JM, Christou-Savina S, Xiong Y, Moede T, Moruzzi N, Karlsson-Edlund P (2014). Ciliary dysfunction impairs beta-cell insulin secretion and promotes development of type 2 diabetes in rodents. Nat Commun.

[68] Song DK, Choi JH, Kim MS (2018). Primary cilia as a signaling platform for control of energy metabolism. Diabetes Metab J.

[69] Zhu D, Shi S, Wang H, Liao K (2009). Growth arrest induces primary-cilium formation and sensitizes IGF-1-receptor signaling during differentiation induction of 3T3-L1 preadipocytes. J Cell Sci.

[70] Koepsell H (2020). Glucose transporters in brain in health and disease. Pflug Arch.

[71] Ren H, Lu TY, McGraw TE, Accili D (2015). Anorexia and impaired glucose metabolism in mice with hypothalamic ablation of Glut4 neurons. Diabetes.

[72] Reno CM, Puente EC, Sheng Z, Daphna-Iken D, Bree AJ, Routh VH (2017). Brain GLUT4 knockout mice have impaired glucose tolerance, decreased insulin sensitivity, and impaired hypoglycemic counterregulation. Diabetes.

[73] Brumfield A, Chaudhary N, Molle D, Wen J, Graumann J, McGraw TE (2021). Insulin-promoted mobilization of GLUT4 from a perinuclear storage site requires RAB10. Mol Biol Cell.

[74] Babbey CM, Bacallao RL, Dunn KW (2010). Rab10 associates with primary cilia and the exocyst complex in renal epithelial cells. Am J Physiol Ren Physiol.

[75] Ayala JE, Samuel VT, Morton GJ, Obici S, Croniger CM, Shulman GI (2010). Standard operating procedures for describing and performing metabolic tests of glucose homeostasis in mice. Dis Model Mech.

[76] Dummer A, Poelma C, DeRuiter MC, Goumans MJ, Hierck BP (2016). Measuring the primary cilium length: improved method for unbiased high-throughput analysis. Cilia.

[77] Schwieger J, Esser KH, Lenarz T, Scheper V (2016). Establishment of a long-term spiral ganglion neuron culture with reduced glial cell number: effects of AraC on cell composition and neurons. J Neurosci Methods.

[78] Rodriguez-Navas C, Morselli E, Clegg DJ (2016). Sexually dimorphic brain fatty acid composition in low and high fat diet-fed mice. Mol Metab.

[79] Bernal-Sore I, Navarro-Marquez M, Osorio-Fuentealba C, Diaz-Castro F, Del Campo A, Donoso-Barraza C (2018). Mifepristone enhances insulin-stimulated Akt phosphorylation and glucose uptake in skeletal muscle cells. Mol Cell Endocrinol.

