# Supplementary material for: Palmitic acid control of ciliogenesis modulates insulin signaling in hypothalamic neurons through an autophagy-dependent mechanism
Source: Cell Death Dis. 2022 Jul 28;13(7):659. doi: 10.1038/s41419-022-05109-9 (PMC9334645; doi:10.1038/s41419-022-05109-9)
Supplement: Supplementary file 1 — Supplementary Information (Figures and experimental procedures) [file 41419_2022_5109_MOESM1_ESM.pdf]

Supplementary Information

Figures and Experimental Procedures

SUPPLEMENTARY FIGURES

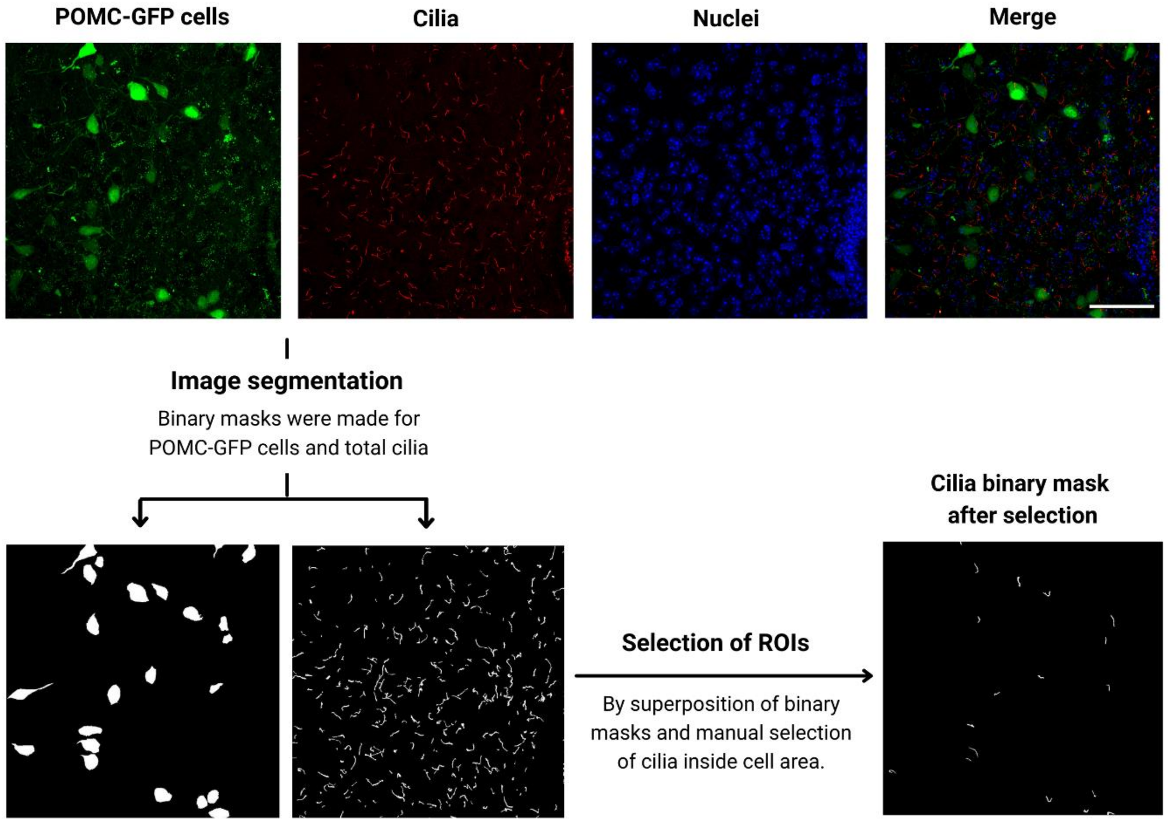

Supplementary Figure 1: Selection of POMC-GFP cell cilia: Representative image showing POMC-eGFP cells (green), primary cilia (red), nuclei (blue) individual channels and the merge image. Z-stacks (n=15; 0.14µm/slice) images have been acquired using Zeiss LSM 880 confocal microscope with Airyscan detection using a 40x objective with 1.2 NA (pixel size 0.08 µm). Image processing was carried out by IDL-based software for automated and curated segmentation of cell area, and focal adhesion morphometrics (https://github.com/KanchanawongLab/Morphometry). Simple or Otsu thresholding were used to generate binary mask of the maximum intensity projections for primary cilia and POMC-eGFP cells. The primary cilia which matched the area of POMC-eGFP cells were determined by superposition of these binary masks, and selected as regions of interest (ROIs), eliminating those outside of the cell's area. Scale bar 100 µm.

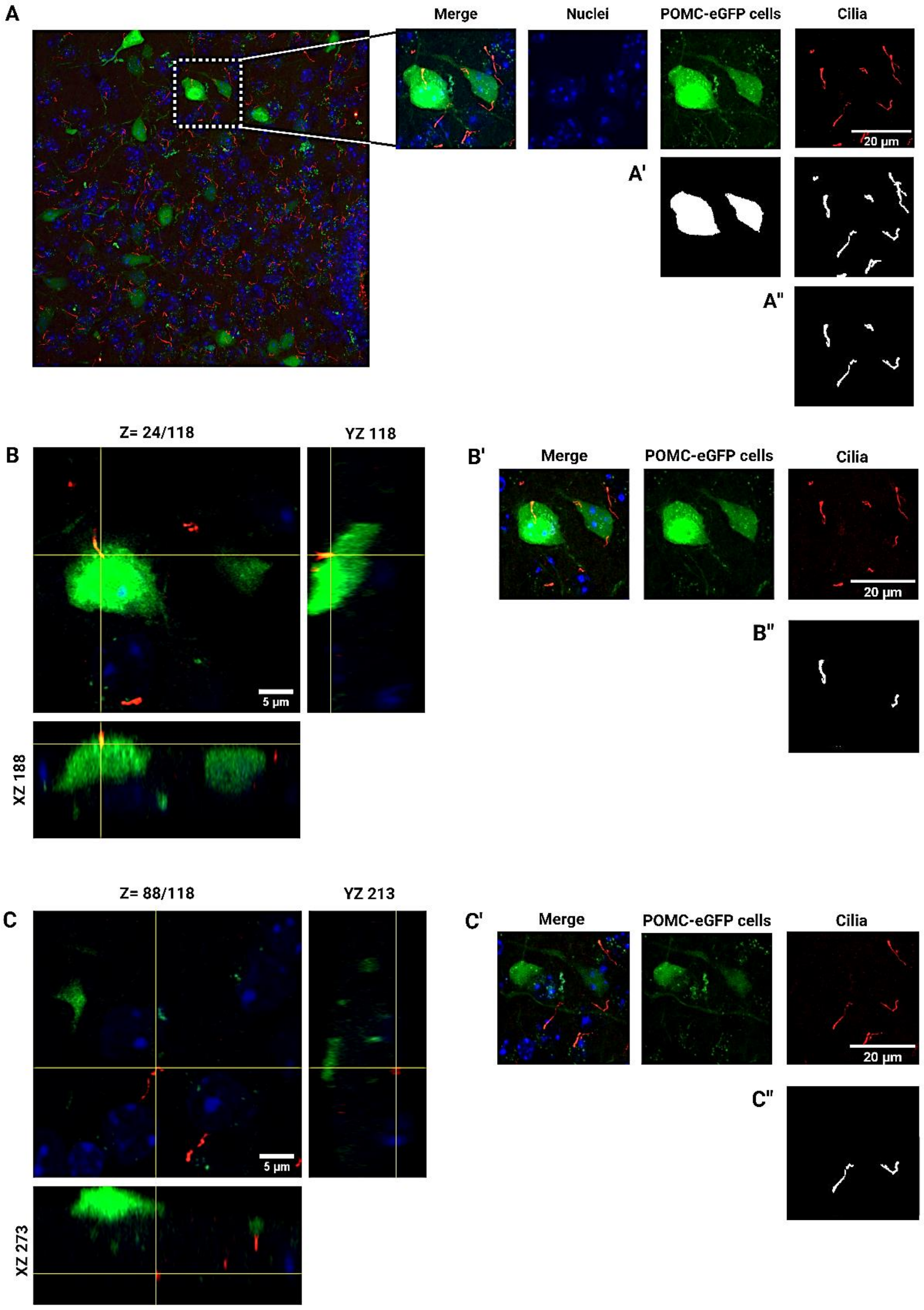

Supplementary Figure 2: Determination of single cilium per POMC cell. **(A)** Example of POMC cell image, as in Supplementary Fig. 1, and zoom image with its individual channels: nuclei (blue), POMC-eGFP cells (green), and primary cilia (red). Binary masks obtained after segmentation of cell and cilia area **(A')** were superposed to select only the cilia

matching the POMC cell's area, obtaining a new binary mask with the selected regions of interest (ROIs) **(A")**. **(B)** Orthogonal views of the zoom-in image, at z= 24/118, showing the colocalization of the primary cilium (red) and the POMC cell (green). **(B')** Maximum intensity projection of z-stacks 1 to 56 (of a total of 118), corresponding to the cilium and POMC cell's localization on the z-axis, and the corresponding binary mask with ROIs found in z-stacks 1-56 (**B"**). Given the colocalization of both cilium and cell, this cilium was determined to belong to the POMC cell in the final selection of ROIs for this image **(B")**. **(C)** Orthogonal views of the zoom-in image, at z= 88/118, showing the localization of another primary cilium (red) selected in the initial image segmentation. **(C')** Maximum intensity projection of z-stacks 60 to 97 (of a total of 118), corresponding to the cilium's localization on the z-axis. **(C")** Binary mask with ROIs found in z-stacks 60-97. Given that this cilium was localized between z= 60-97, whereas its allegedly corresponding POMC cell localizes between z= 1-56, this cilium was determined to not belong to the POMC cell in the final selection of ROIs for this image. Distance between z-stacks = 0.14 μm/slice.

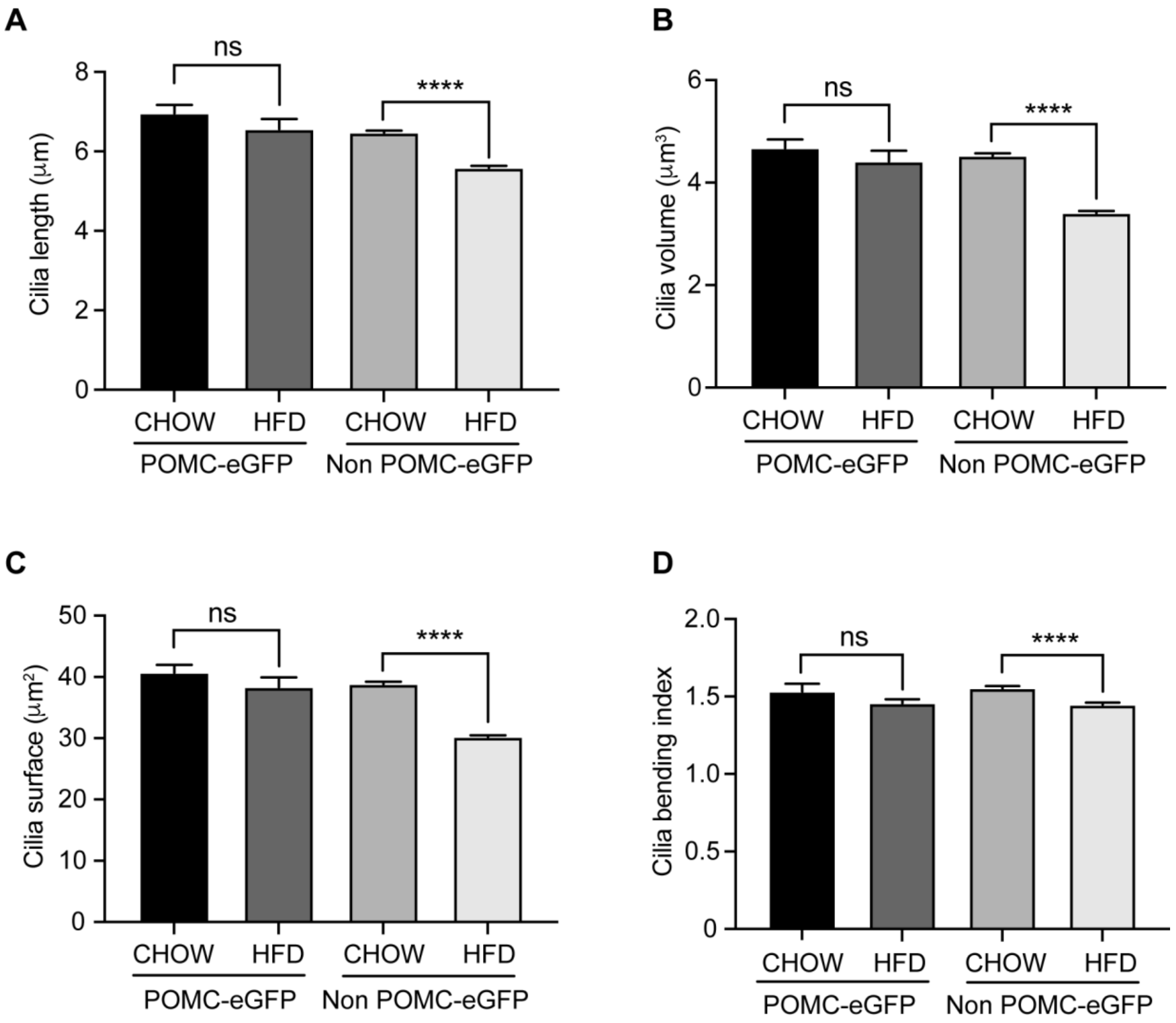

Supplementary Figure 3: Quantification of **(A)** cilia length, **(B)** cilia volume, **(C)** cilia surface and, **(D)** cilia bending index of POMC-eGFP and non POMC-eGFP neurons from mice fed chow or high fat diet (HFD) for 16 weeks. Data are presented as mean ± SEM. Statistical differences were evaluated by using One-way ANOVA followed by post hoc Tukey's multiple comparison test. ****$p < 0.0001$. ns, not significant. n = 4/group.

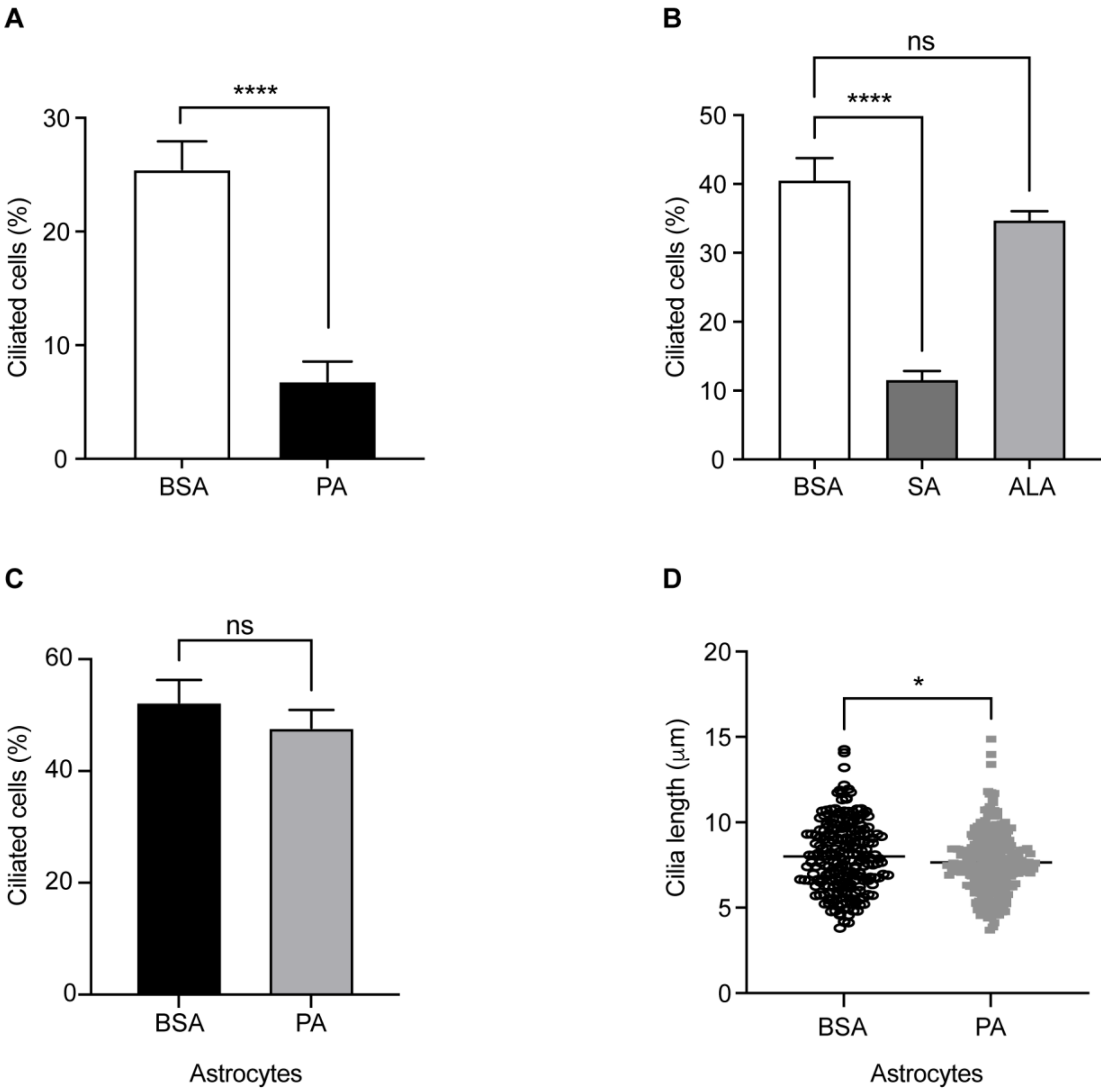

Supplementary Figure 4: Quantification of the percentage of ciliated N43/5 hypothalamic neuronal cells treated with **(A)** BSA or palmitic acid (PA, 100 μM) for 6h, or following 6h of **(B)** BSA, stearic acid (SA, 100 μM) or α-Linolenic acid (ALA, 100 μM) exposure. After fixation, cells were stained against acetylated α-tubulin to identify the ciliary axoneme. Quantification of **(C)** percentage of ciliated cells and **(D)** cilia length of primary hypothalamic astrocytes treated with BSA or PA (100 μM) for 6h. Data are presented as mean ± SEM. Comparisons between two conditions were made using the unpaired two-tailed Student t-test. One-way ANOVA was used for comparison of more than 2 groups, followed by Tukey's post hoc adjustment. $*p < 0.05$, $****p < 0.0001$. ns, not significant. $n = 3$.

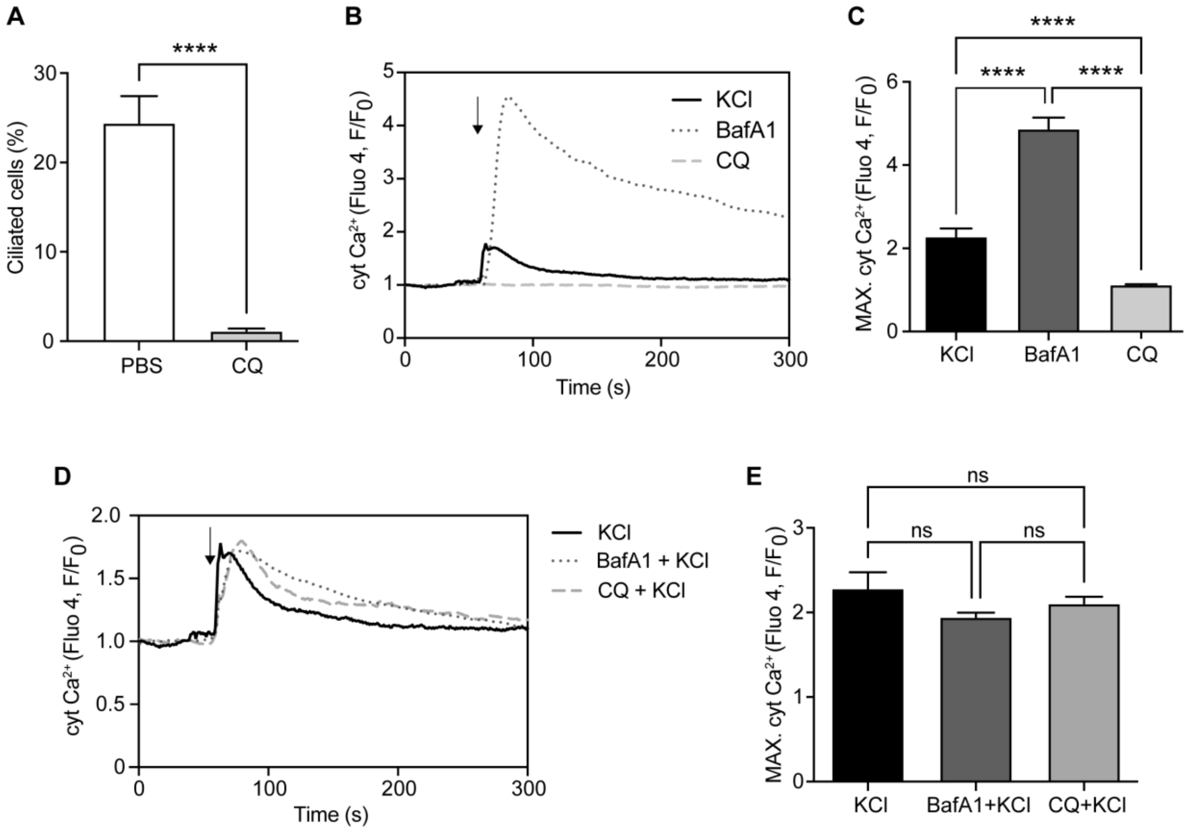

Supplementary Figure 5: (A) Ciliated cells percentage of N43/5 hypothalamic cells treated with PBS (control) or chloroquine (CQ) 30 µM for 6 h. **(B)** Intracellular Ca^{2+} levels of N43/5 cells stimulated with 50 mM KCL (positive control of intracellular Ca^{2+} levels increase), Bafilomycin A1 (BafA1, 100 nM) or CQ (30 µM), with the quantifications **(C)**. **(D)** Intracellular Ca^{2+} levels of N43/5 cells treated with BafA1 (100 nM) or CQ (30 µM) for 6 h followed by stimulation with KCL (50 mM), with the quantification **(E)**. For more details, see supplementary experimental procedures section. Data are presented as mean ± SEM. Comparisons between two conditions were made using the unpaired two-tailed Student t-test. One-way ANOVA was used for comparison of more than 2 groups, followed by Tukey's post hoc adjustment. ****$p < 0.0001$. ns, not significant. n = 3.

SUPPLEMENTARY VIDEOS LEGENDS

Supplementary Video 1: Animated 3D projection of representative crop image seen in **Suppl. Figure 2**, rotating on the Y-axis, and showing the z-localization of POMC-eGFP cells (green), nuclei (blue) and primary cilia (red). 3D reconstruction was performed using the complete z-stack, of all three channels, consisting of 118 images (0.14um/slice) obtained using a LSM 880 Zeiss inverted confocal microscope with Airyscan detection, using a 40x objective with 1.2 NA.

Supplementary Video 2: Animation displaying z-stack scanning, from bottom to top, of representative crop image seen in **Suppl. Figure 2**, showing the z-localization of POMC-eGFP cells (green), nuclei (blue) and primary cilia (red). Stack consists of a total 118 images (0.14um/slice) obtained using a LSM 880 Zeiss inverted confocal microscope with Airyscan detection, using a 40x objective with 1.2 NA.

SUPPLEMENTARY EXPERIMENTAL PROCEDURES

Selection and determination of single cilium per POMC-eGFP cell

For each image, 15 z-stacks were taken for each treatment (Chow and HFD) in 3 different channels: nuclei (405 nm), primary cilia (568 nm) and POMC-eGFP cells (488 nm). Of each stack, a single merge was obtained of the maximum intensity projection. Using an IDL-based software, Morphometry, the maximum intensity projection of each image was segmented, obtaining binary masks for nuclei, primary cilia and POMC-eGFP cells of each image. The primary cilia which matched the area of POMC-eGFP cells were determined by superposition of these binary masks, and selected as regions of interest (ROIs), eliminating those outside of the cell's area. To then determine which of these ROIs indeed corresponded to each POMC-eGFP cell, as only one primary cilium would be expected per cell[1], the z-plane in which each marked POMC-eGFP was found would then be closely analyzed, to confirm which of the cilia identified in the binary mask superposition matched the cell's position, thus also eliminating the cilium that didn't correspond to the same plane or to the cell's area. In the case of the cell at the top left corner of Figure 1E, for instance, both the cell and the corresponding cilium (marked with white arrowhead) where localized between z= 1-60, whereas the cilium marked with the gray arrowhead was localized in z=60-96, and did not match the cell's position (see Supplementary Fig. 2, and Suppl. videos S1 and S2).

Primary culture of hypothalamic astrocytes

Primary cultures of hypothalamic astrocytes were prepared from 0-2 day old C57BL/6 pups. Pups were euthanized by decapitation, and the hypothalami were dissected free of

extraembryonic membranes using a magnifying glass and digested with DNAse (D4263, Sigma-Aldrich, St. Louis, MO, USA) and trypsin-EDTA (5400054, Gibco, USA) in HBSS (14185052, Gibco) for 15 minutes, 37°C. Tissues were then washed and dissociated using high-glucose Dulbecco's Modified Eagle's Medium (DMEM; D2902, Gibco) supplemented with 10% horse serum (adhesion medium). Next, the suspension was centrifuged for 5 min, 3500 rpm, 4 °C and cells were grown in culture flasks with this medium for 24 h and maintained at 37 °C with 5% CO_2. Then, astrocyte primary cultures were maintained in DMEM supplemented with 10% FBS (16000044, Gibco) and penicillin/streptomycin (15140122, Gibco) at 37 °C with 5% CO_2. The medium was changed 24 h before fatty acid treatments to DMEM high glucose supplemented with 2% FBS and penicillin/streptomycin.

Cell line culture and treatments

N43/5 cells (Cellutions Biosystems, Canada) were cultured in high glucose Dulbecco's modified eagle medium (HG-DMEM) (11995-040, Gibco, USA) supplemented with 10% fetal bovine serum (FBS) (10437028, Gibco), 100 U/ml penicillin-streptomycin (15140122, Gibco) and maintained at 37 °C with 5% CO2. Cells were incubated with HG-DMEM supplemented with 2% FBS 24 h before treatments. Then, cells were exposed to 100 µM palmitic acid (PA; P0500, Sigma-Aldrich), 100 µM stearic acid (SA; S4751, Sigma-Aldrich) or 100 µM α-Linolenic acid (ALA; L2376, Sigma-Aldrich) conjugated to fatty acid-free bovine serum albumin (BSA) (152401, MP Biomedicals, Santa Ana, CA, USA) for 6 h. BSA treatment was used as control. In order to assess the changes in the primary cilium after the inhibition of the autophagic flux, cells were treated with Chloroquine (CQ; C6628 Sigma-Aldrich) at concentrations of 30 µM for 6 h or with PBS (vehicle).

Measurement of intracellular Ca^{2+} levels

Cellular Ca^{2+} images were obtained from cells preloaded with Fluo4 (fluo4-AM, Molecular Probes, 5 mM, 30 min) using an inverted confocal microscope (Carl Zeiss LSM 5, Pascal 5 Axiovert 200 microscope), as described previously [2, 3]. Briefly, cell-containing coverslips were mounted in a 1 ml capacity chamber and placed in the microscope for confocal measurements after excitation with a laser line (excitation 488 nm; emission 526 nm). KCl (50 mM), used as positive control since it increases intracellular Ca^{2+} levels in neuronal cells [4], bafilomycin A1 (BafA1, 100 nm), chloroquine (CQ, 30 µm) and palmitic acid (PA, 100 µm) were either added directly or the solution was rapidly exchanged in the camera (1 s) at 50 s. Fluorescent images were collected every 0.4–2.0 s for fast signals and analyzed frame by frame with the Image J software (NIH, Bethesda, MD). Intracellular Ca^{2+} levels are expressed as relative fluorescence, DF/F_0, where DF represents the difference between the experimental value F and the basal fluorescence value F_0. Within the range defined for each probe, the fluorescence intensity increases proportionally with intracellular Ca^{2+} concentration[5]. Experimental determinations were carried out in cells bathed with Ca^{2+}-containing Krebs buffer (140 mM NaCl, 5 mM KCl, 1 mM $CaCl_2$, 1 mM $MgCl_2$, 10 mM HEPES, 1 mg/ml glucose).

REFERENCES

1. Ostrowski LE, Dutcher SK, Lo CW. Cilia and models for studying structure and function. *Proc Am Thorac Soc* 2011, **8**(5)**:** 423-429.

2. Chiong M, Parra V, Eisner V, Ibarra C, Maldonado C, Criollo A*, et al.* Parallel activation of Ca(2+)-induced survival and death pathways in cardiomyocytes by sorbitol-induced hyperosmotic stress. *Apoptosis* 2010, **15**(8)**:** 887-903.

3. Parra V, Altamirano F, Hernandez-Fuentes CP, Tong D, Kyrychenko V, Rotter D*, et al.* Down Syndrome Critical Region 1 Gene, Rcan1, Helps Maintain a More Fused Mitochondrial Network. *Circ Res* 2018, **122**(6)**:** e20-e33.

4. Imanishi T, Matsushima K, Kawaguchi A, Wada T, Yoshida S, Ichida S. Increased response to high KCl-induced elevation in the intracellular-Ca(2+) concentration in differentiated NG108-15 cell and the inhibitory effect of the L-type Ca(2+) channel blocker, calciseptine. *Neurochem Res* 2006, **31**(1)**:** 33-40.

5. Ibarra C, Estrada M, Carrasco L, Chiong M, Liberona JL, Cardenas C*, et al.* Insulin-like growth factor-1 induces an inositol 1,4,5-trisphosphate-dependent increase in nuclear and cytosolic calcium in cultured rat cardiac myocytes. *J Biol Chem* 2004, **279**(9)**:** 7554-7565.
