# Supplementary figures and images for: Palmitic acid control of ciliogenesis modulates insulin signaling in hypothalamic neurons through an autophagy-dependent mechanism
Source: Cell Death Dis. 2022 Jul 28;13(7):659. doi: 10.1038/s41419-022-05109-9 (PMC9334645; doi:10.1038/s41419-022-05109-9)

# Supplementary Material

## Western Blots

**Fig. 2**

**E**

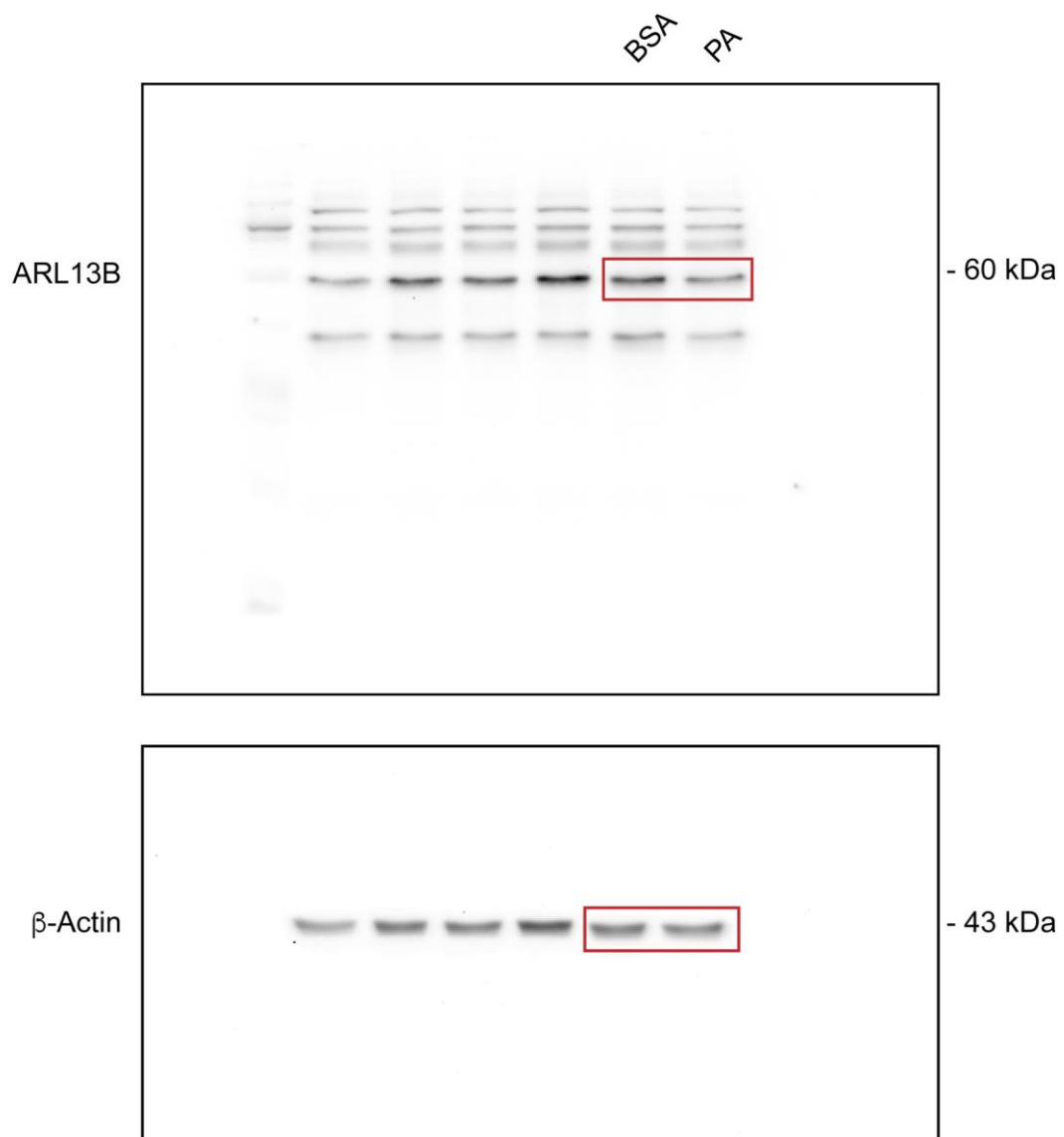

**Fig. 3**

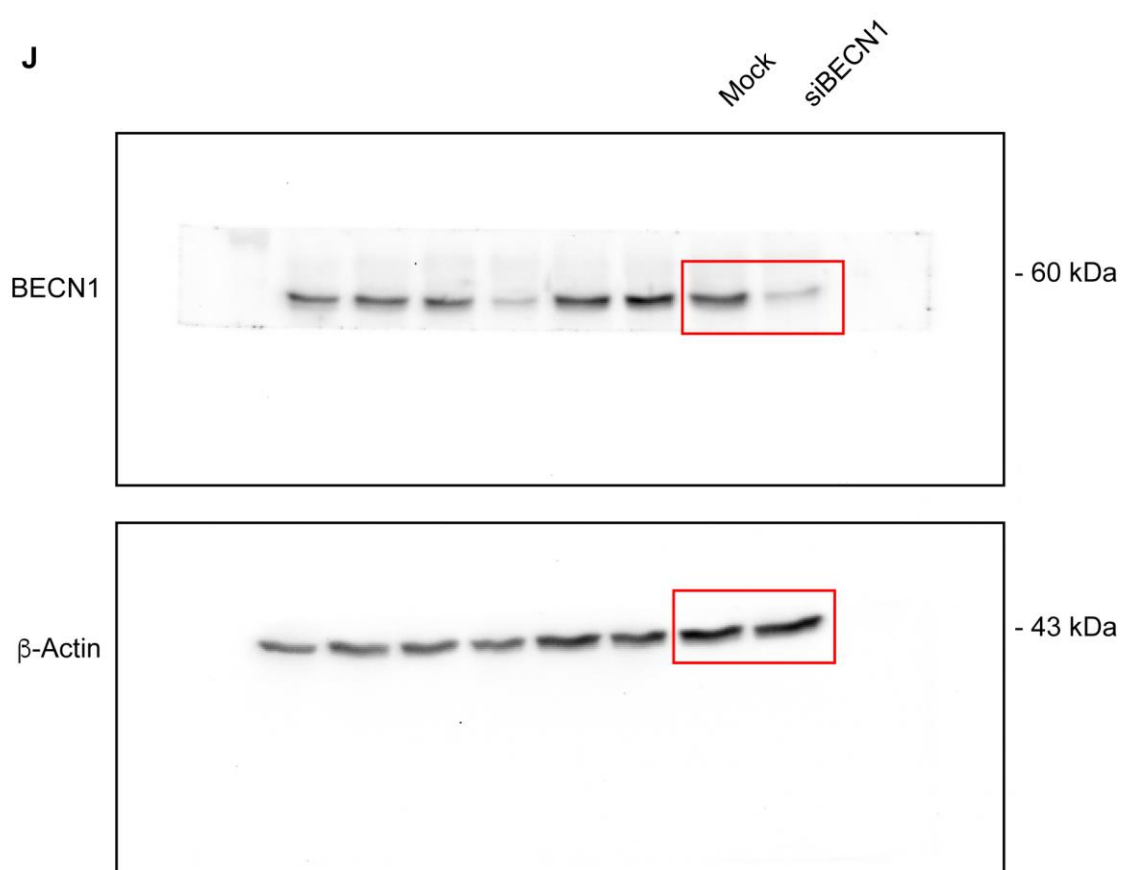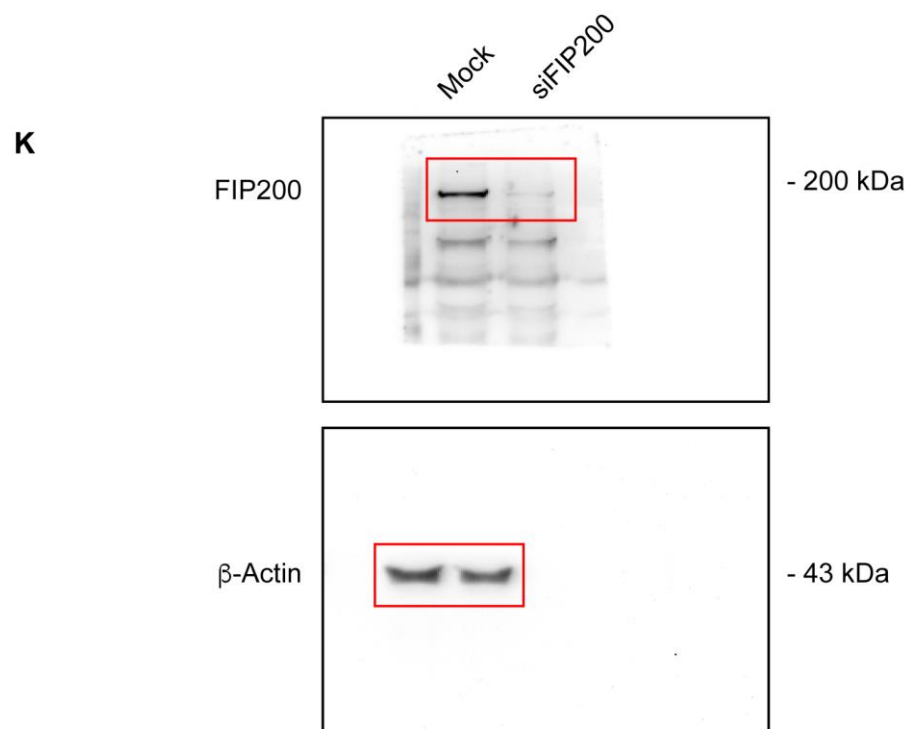

**Fig. 4**

**C**

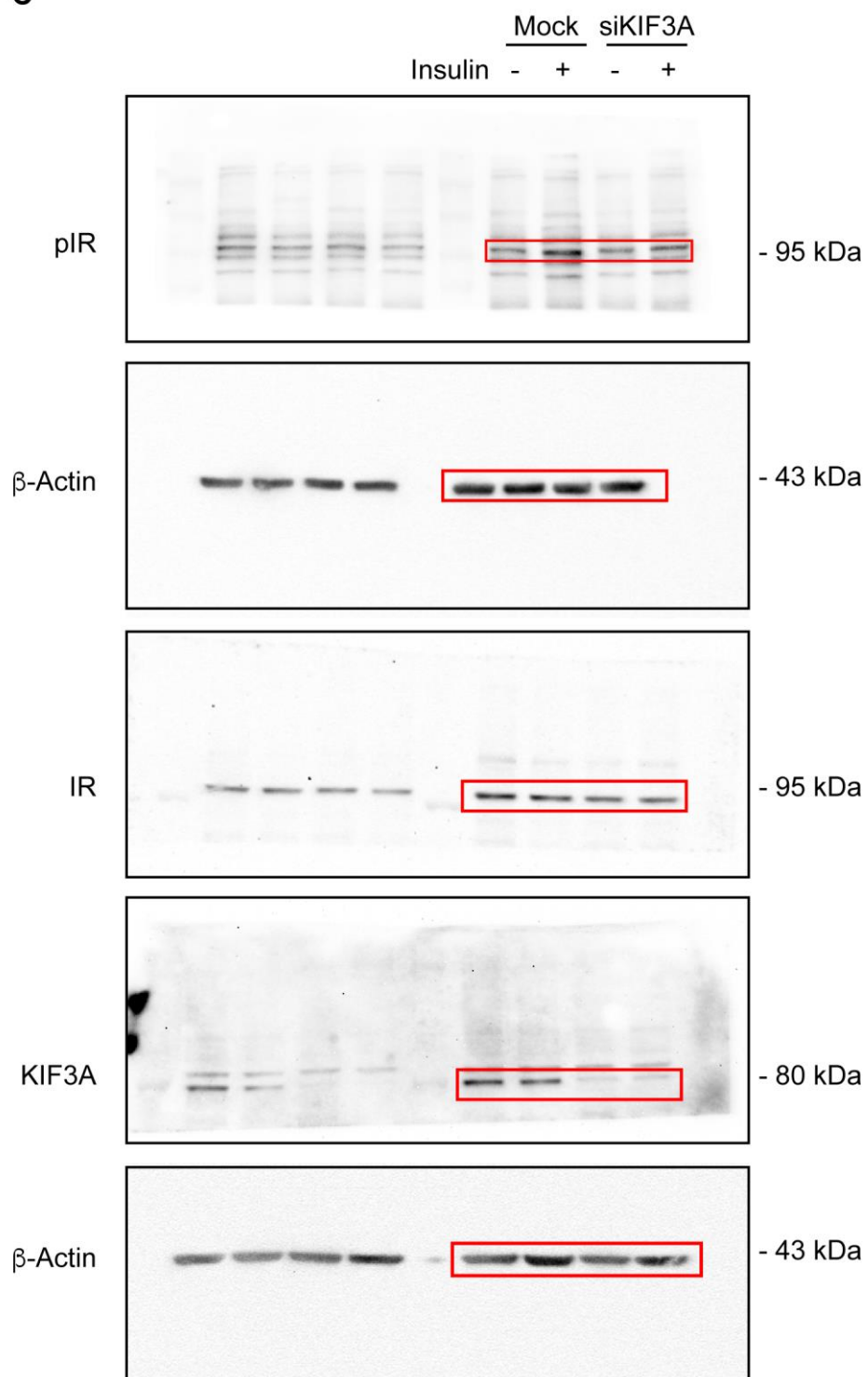

**Fig. 4**

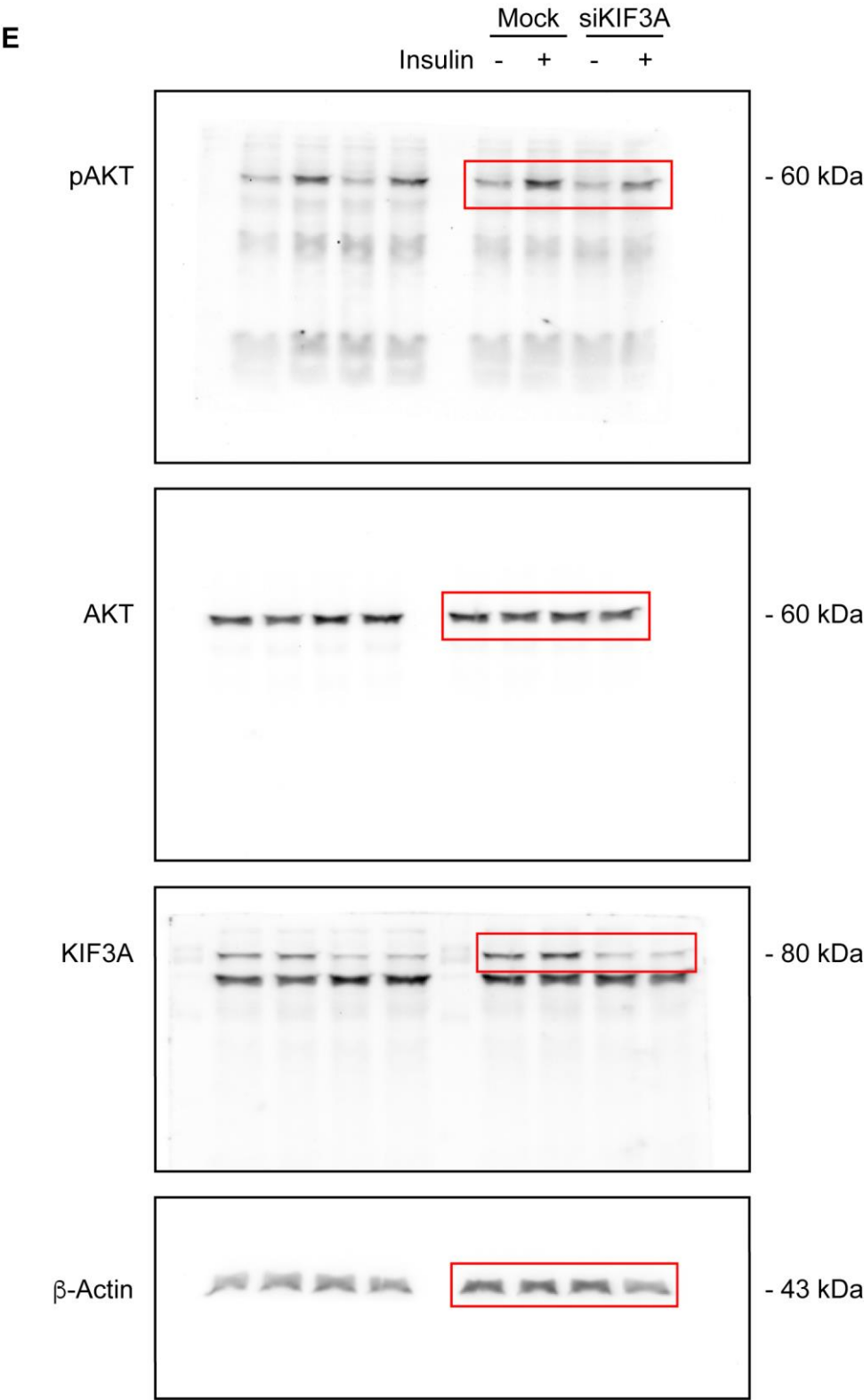

**Fig. 4**

**H**

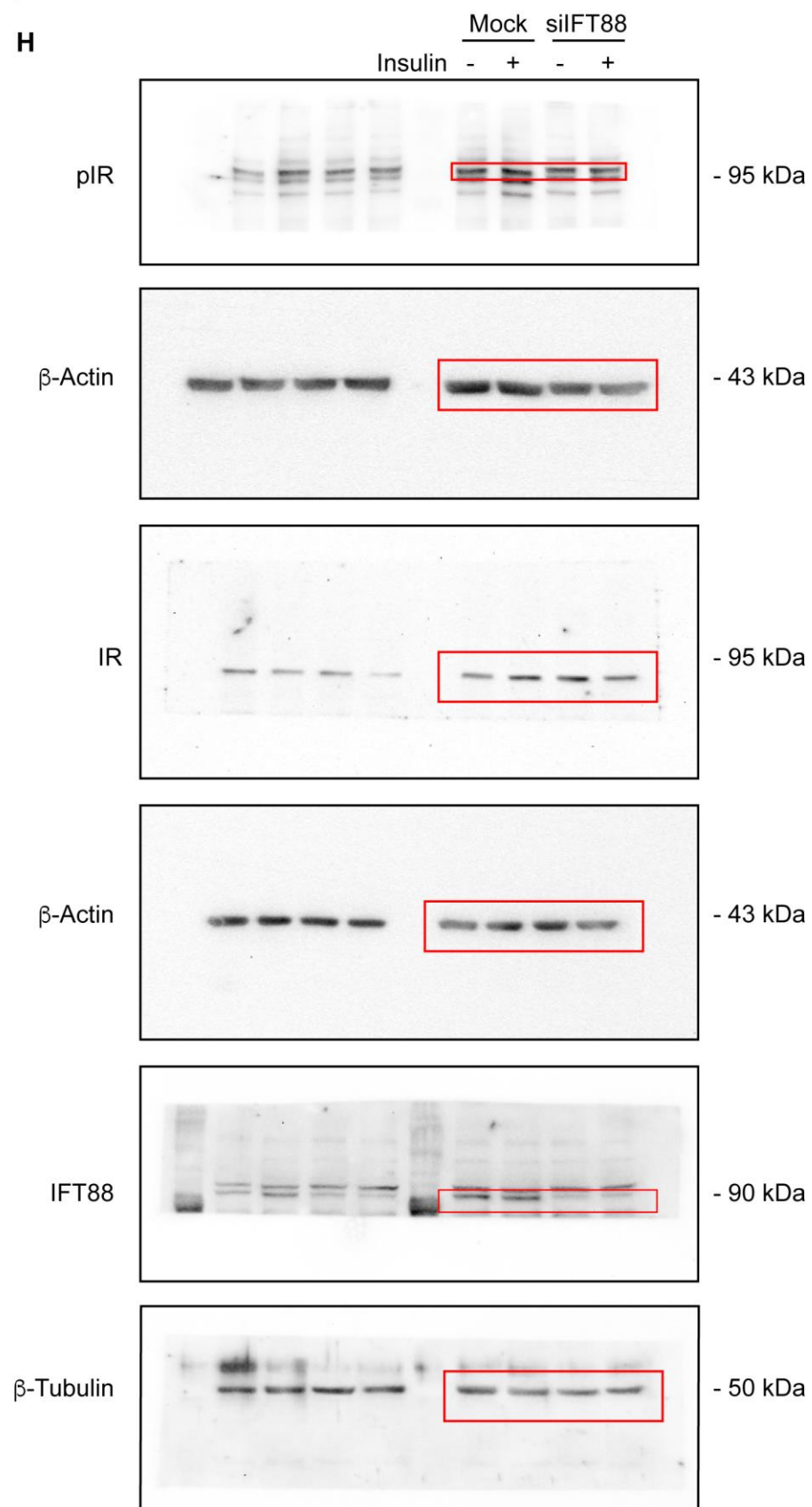

**Fig. 4**

**L**

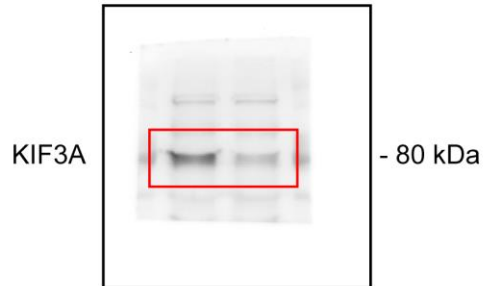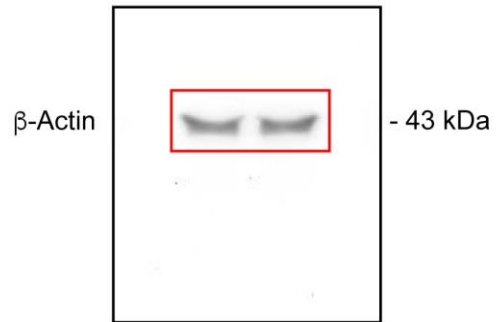

**M**

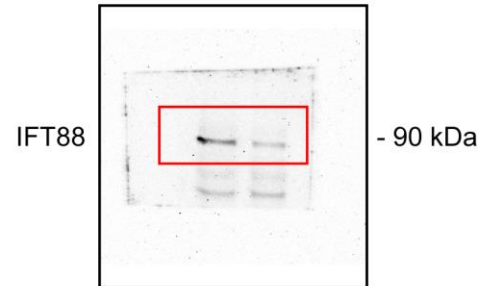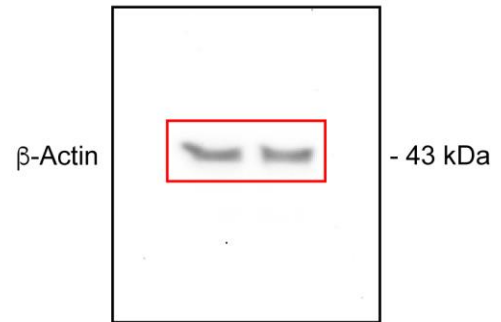

**Fig. 5**

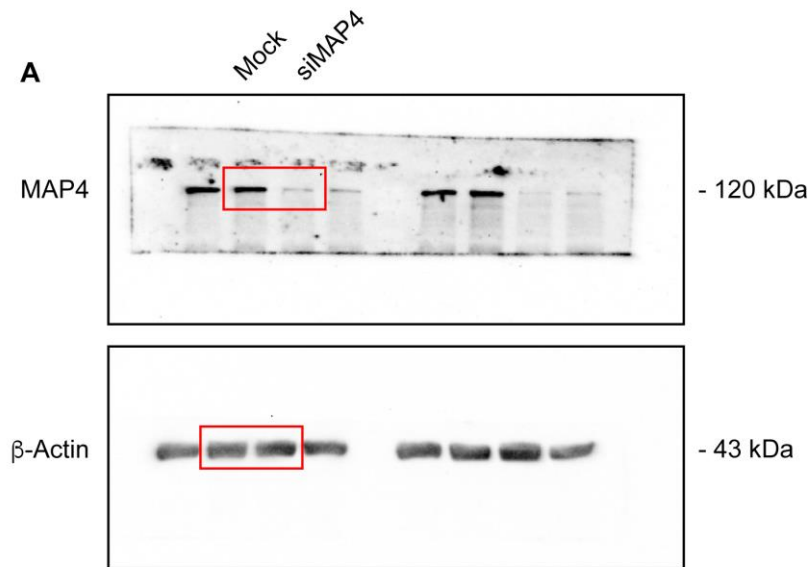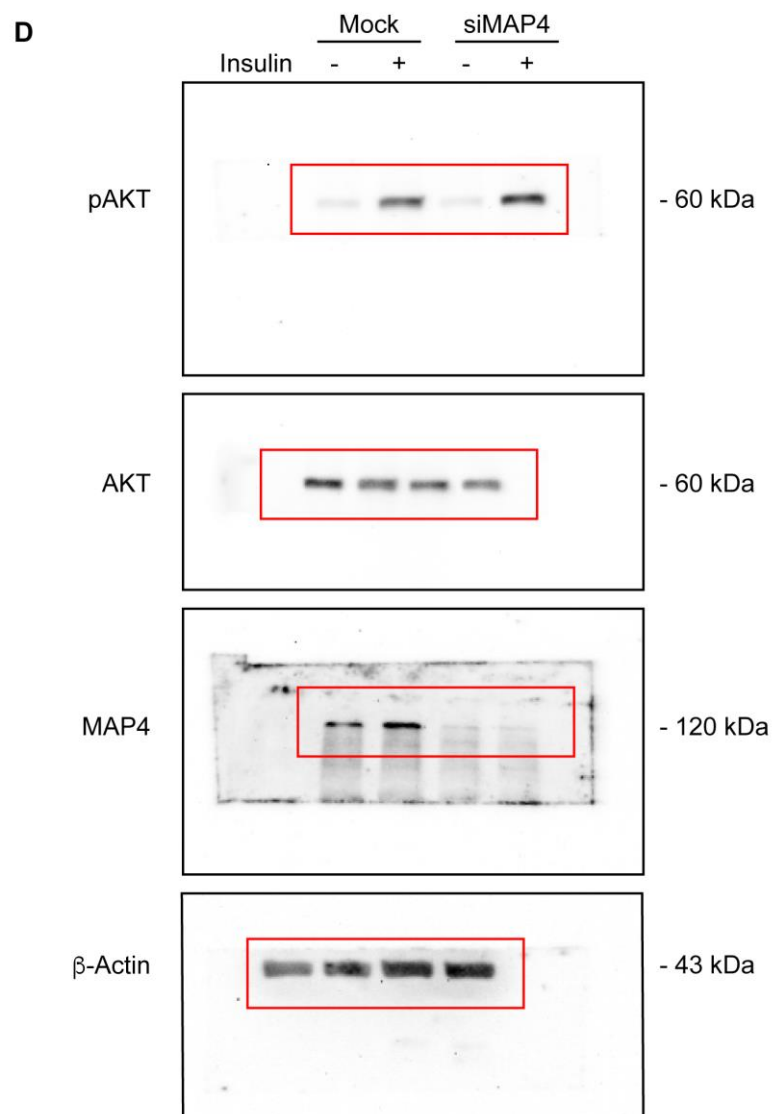

Fig. 5

I

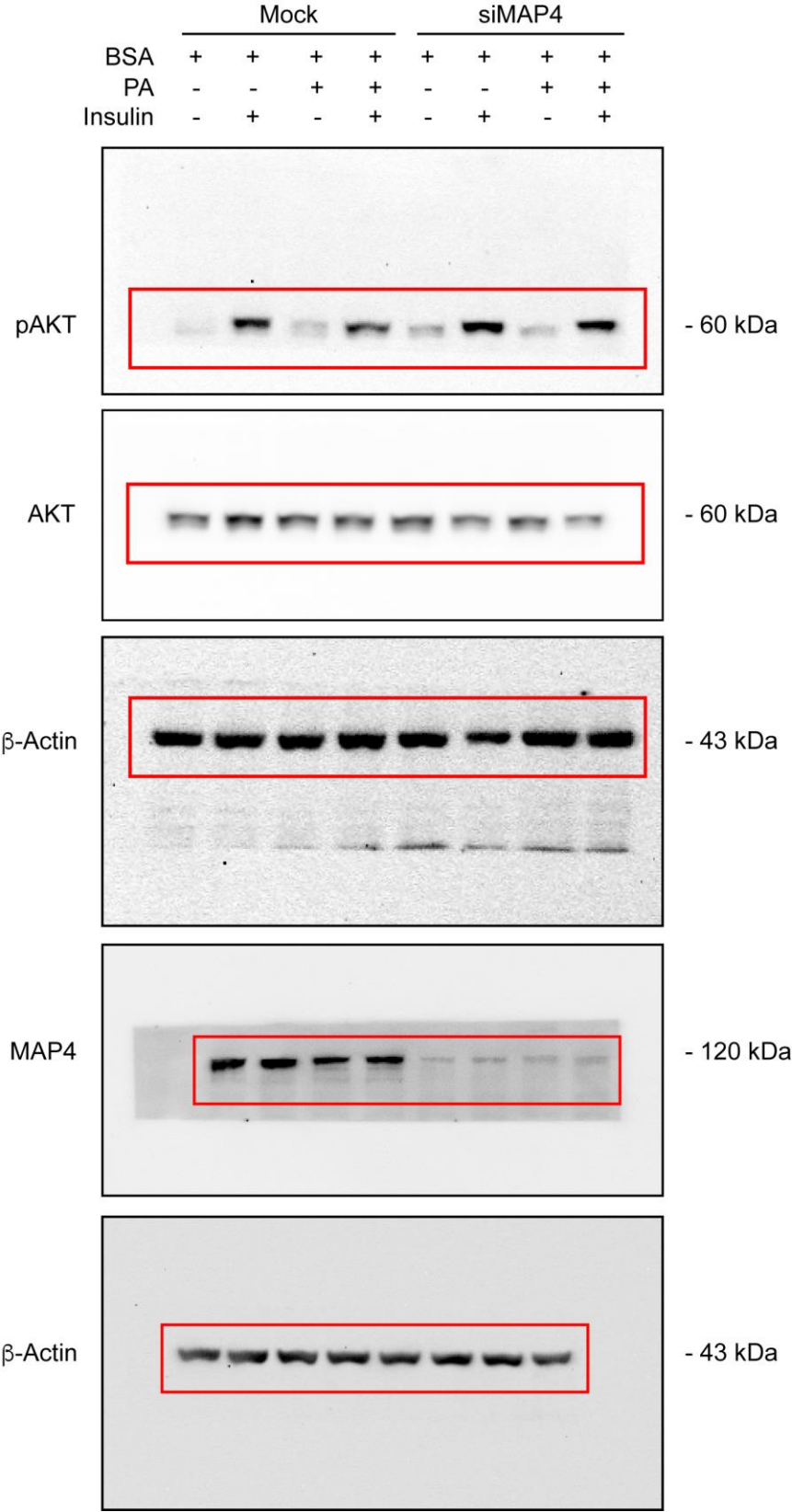

Supplement: Supplementary file 4 — Supplementary Material - Western Blots [file 41419_2022_5109_MOESM4_ESM.pdf]
